# RecurIndex-Guided postoperative radiotherapy with or without Avoidance of Irradiation of regional Nodes in 1–3 node-positive breast cancer (RIGAIN): a study protocol for a multicentre, open-label, randomised controlled prospective, phase III trial

**DOI:** 10.1136/bmjopen-2023-078049

**Published:** 2024-07-30

**Authors:** Jing Liu, Yuting Tan, Zhuofei Bi, Suning Huang, Na Zhang, An-du Zhang, Lina Zhao, Yu Wang, Zibin Liang, Yu Hou, Xiangying Xu, Jianying Chen, Fei Wang, Xiaowen Lan, Xiao Lin, Xiaoxue Zhang, Wenyi Zhou, Xuting Ye, Jian-gui Guo, Xiaohong Wang, Ran Ding, Jiayi Chen, Xiaobo Huang

**Affiliations:** 1 Department of Radiation Oncology, Sun Yat-Sen Memorial Hospital, Sun Yat-Sen University, Guangzhou, Guangdong, China; 2 Department of Radiotherapy for Breast Tumor, Yat-Sen Breast Tumor Hospital, Sun Yat-Sen Memorial Hospital, Sun Yat-Sen University, Guangzhou, Guangdong, China; 3 Department of Radiotherapy, Guangxi Medical University Cancer Hospital, Nanning, Guangxi Zhuang Autonomous Region, China; 4 Department of Radiation Oncology, Liaoning Cancer Hospital & Institute, Shenyang, Liaoning, China; 5 Department of Radiation Oncology, The fourth hospital of hebei medical university, Shijiazhuang, Hebei, China; 6 Department of Radiation Oncology, Xijing Hospital, Xian, Shaanxi, China; 7 Department of Radiation Oncology, Shanxi Provincial Cancer Hospital, Taiyuan, Shanxi, China; 8 Department of Thoracic Oncology, The Fifth Affiliated Hospital of Sun Yat-Sen University, Zhuhai, Guangdong, China; 9 Department of Radiation Oncology, Peking University Cancer Hospital Yunnan / Yunnan Cancer Hospital / The Third Affiliated Hospital of Kunming Medical University, Kunming, Yunnan, China; 10 Department of Radiation Oncology, Third Affiliated Hospital of Sun Yat-Sen University, Guangzhou, Guangdong, China; 11 State Key Laboratory of Neurology and Oncology Drug Development, Jiangsu Simcere Pharmaceutical Research Company, Nanjing, Jiangsu, China; 12 Department of Breast Oncology, The First People’s Hospital of Foshan, Foshan, Guangdong, China; 13 The sixth department of chemoradiotherapy, Tangshan People's Hospital, Tangshan, Hebei, China; 14 Department of Radiation Oncology, Ruijin Hospital, Shanghai Jiaotong University School of Medicine, Shanghai, China

**Keywords:** RADIOTHERAPY, Breast tumours, Oncogenes

## Abstract

**ABSTRACT:**

**Introduction:**

Postoperative radiotherapy in patients with breast cancer with one to three lymph node metastases, particularly within the pT1–2N1M0 cohort with a low clinical risk of local–regional recurrence (LRR), has incited a discourse surrounding personalised treatment strategies. Multigene testing for Recurrence Index (RecurIndex) model capably differentiates patients based on their level of LRR risk. This research aims to validate whether a more aggressive treatment approach can enhance clinical outcomes in N1 patients who possess a clinically low risk of LRR, yet a high RecurIndex-determined risk of LRR. Specifically, this entails postoperative whole breast irradiation combined with regional lymph node irradiation (RNI) following breast-conserving surgery or chest wall irradiation with RNI after mastectomy.

**Methods and analysis:**

The RIGAIN (RecurIndex-Guided postoperative radiotherapy with or without Avoidance of Irradiation of regional Nodes in 1–3 node-positive breast cancer) Study is a multicentre, prospective, randomised, open-label, phase III clinical trial that is being conducted in China. In this study, patients with low clinical LRR risk but high RecurIndex-LRR risk are randomly assigned in a 1:1 ratio to the experimental group or the control group. In the experimental group, RNI is performed and the control group omits RNI. Efficacy and safety analyses will be conducted, enrolling a total of 540 patients (270 per group). The primary endpoint is invasive disease-free survival, and secondary endpoints include any first recurrence, LRR-free survival, distant metastasis-free survival, recurrence-free survival, overall survival, disease-free survival, breast cancer-specific mortality and assessment of patient quality of life. The study began in April 2023 and with a follow-up period of 60 months after the last participant completes radiation therapy.

**Ethics and dissemination:**

The study was approved by the Ethics Committee of Sun Yat-Sen Memorial Hospital, Sun Yat-Sen University (SYSKY-2022-097-02, V.3.1). It adheres to the Helsinki Declaration and Good Clinical Practice. Research findings will be submitted for publication in peer-reviewed journals.

**Trial registration number:**

NCT04069884.

**STRENGTHS AND LIMITATIONS OF THIS STUDY**

- The trial is designed as a multicentre, open-label, randomised controlled, phase III study.
- Introduced a multigene model to guide precision radiotherapy.
- This research uses invasive disease-free survival as the primary endpoint.
- The trial is conducted only in one country (China).

## Introduction

 Patients with one to three axillary lymph node metastases constitute approximately 25–30% of early operable breast cancer cases. Radiotherapy plays a pivotal role in the comprehensive treatment of breast cancer.^1 2^ However, the benefit of postoperative radiotherapy for patients with N1 breast cancer, particularly in terms of survival improvement, remains a topic of substantial debate. Studies conducted in the 1990s such as the Vancouver study, DBCG-82b/82c, and the early meta-analysis by the Early Breast Cancer Trialists’ Collaborative Group (EBCTCG) (including the N1 subgroup) consistently demonstrated that postoperative radiotherapy significantly enhances disease-free survival (DFS) and overall survival (OS) for patients.^3^,^7^ Consequently, N1 becomes a relative indication for postoperative radiotherapy. The 2011/2014 EBCTCG meta-analysis further suggested that postoperative radiotherapy could convert a 1.5% reduction in the 10-year any first recurrence (AFR) rate into a 1% 20-year OS benefit.^8 9^ The MA20 and European Organization for Research and Treatment of Cancer (EORTC) 22922 studies published in 2015,^10 11^ which focused on T1–2N1 patients, especially those with high clinical risk of local–regional recurrence (LRR) and compared postoperative regional lymph node irradiation (RNI) after breast-conserving surgery (BCS) or without RNI, found that more aggressive postoperative RNI for T1–2N1 patients could result in better distant metastases-free survival (DMFS), DFS or breast cancer-specific mortality (BCSM). The Vancouver study’s 20-year long-term follow-up results demonstrated the long-term OS benefit of postoperative radiotherapy in the N1 subgroup. These milestone studies further reinforced the value and recommendation of postoperative radiotherapy for patients with N1 breast cancer, rendering N1 staging a strong relative indication for postoperative radiotherapy and increasing the number of patients actively accepting postoperative radiotherapy. Nevertheless, not all N1 patients will benefit from postoperative radiotherapy. Some real-world retrospective studies reveal limited LRR and/or survival improvement from postoperative radiotherapy, particularly RNI therapy, among certain N1 patients, especially those with relatively low clinical risk. Consequently, the necessity of radiotherapy for clinically low LRR risk N1 patients remains a topic of significant controversy and uncertainty. Clinical practice often presents varying professional recommendations for postoperative radiotherapy in low-risk N1 patients, resulting in the exclusion of a substantial number of patients solely based on traditional clinical and pathological features. However, this omission of RNI could lead to inadequate treatment, with potential implications for tumour recurrence, metastasis and patient survival. Conversely, a uniform approach of postoperative radiotherapy for all clinically low LRR risk N1 patients would inevitably result in overtreatment and expose patients to additional risks such as radiation-induced injury and related complications, thereby impacting their quality of life.^12^,^15^

When considering low-risk patients with N1 breast cancer, then, the primary objective is to identify the actual high-risk patients concealed within the clinically low-risk population and to strategically administer postoperative radiotherapy. This represents one of the essential development directions of early breast cancer ‘precision radiotherapy’ in the future. Achieving individualised and precise radiotherapy depends on the discovery of molecular genetic prediction models that can accurately predict LRR risk in a scientific, reliable and accessible manner. Currently approved multigene detection models abroad include Oncotype DX, MammaPrint and EndoPredict. Oncotype DX is the most representative and extensively used multigene prognostic analysis method, primarily employed to guide early luminal low-risk patients to avoid adjuvant chemotherapy. Oncotype DX is currently more frequently used in the radiotherapy field to identify low-risk elderly N0 breast-conserving patients exempt from postoperative radiotherapy. Although the predictive value in the N1 population has initially demonstrated some clinical significance, contradictions exist between various research findings.^16^,^19^ No prospective high-level evidence for multigene models predicting RNI benefits in N1 patients is currently available. The clinical trial Tailor RT (MA39) conducted by the Canadian Cancer Trials Group primarily investigates whether low-risk recurrence patients can be spared from postoperative radiotherapy or RNI. This is currently the only prospective, randomised controlled, phase III study internationally that uses a multigene predictive model to guide precise radiotherapy for N1 patients. The future research outcomes will primarily be applied to guide the omission of postoperative radiotherapy in clinically low LRR risk and genetically low-risk N1 patients. However, the significance of postoperative radiotherapy for patients with intersecting risks, especially those with clinically low-risk but genetically high-risk profiles, remains uncertain.

Recurrence Index (RecurIndex) is the only risk prediction model developed based on the Chinese population for early-stage breast cancer. Consisting of 18 core genes and 10 immunohistochemical 4 reference genes, it is capable of independently predicting the risk of LRR and distant metastasis.^20^,^24^ Internal validation studies in Taiwan and external validation studies conducted in Singapore, Hong Kong and the Fourth Affiliated Hospital of Hebei Medical University in China have all provided strong evidence of RecurIndex’s predictive efficacy and its value in guiding radiotherapy for N1 patients.^25 26^ Low-risk and high-risk patients identified by RecurIndex-LRR had 5-year LRR rates of 0% and 7%, respectively (p=0.0146). Compared with high-risk RecurIndex-LRR patients who did not receive postoperative radiotherapy, those who underwent postoperative radiotherapy demonstrated significantly improved rates of LRR and recurrence-free survival (RFS), with percentages of 88.8% vs 74.1% (p=0.0071) and 79.4% vs 59.5% (p=0.0019), respectively. These results clearly indicate the significant benefits of postoperative radiotherapy in this patient population. To date, RecurIndex has become widely recognised and clinically implemented around the Asia-Pacific region. It has been incorporated into the ‘Expert Consensus on Multigene Testing for Adjuvant Therapy of Hormone Receptor-Positive, Human Epidermal Growth Factor Receptor 2-Negative Early Breast Cancer’ in China and recommended in the ‘Chinese Society of Clinical Oncology Guidelines for the Diagnosis and Treatment of Breast Cancer 2022’ for guiding precise postoperative radiotherapy in N1 patients. However, further high-level, randomised controlled, phase III clinical trials are needed to validate its clinical applications and expand its usage in the field. The most promising and crucial area for its application lies in guiding precise radiotherapy for patients with N1 breast cancer.

In summary, we have begun conducting a multicentre, prospective, randomised controlled, phase III clinical study of individualised precision radiotherapy for patients with clinically low LRR risk breast cancer with N1 guided by RecurIndex. This study aims to evaluate patients’ local recurrence and distant metastasis risks, primarily investigating whether active postoperative radiotherapy can further improve clinical efficacy in N1 patients with clinically low risk but high RecurIndex-LRR risk. The ultimate goal of this study is to provide high-level clinical evidence and reliable multigene recurrence risk prediction models to help achieve individualised precision radiotherapy for patients with N1 breast cancer.

## Materials and methods

**Figure 1:**
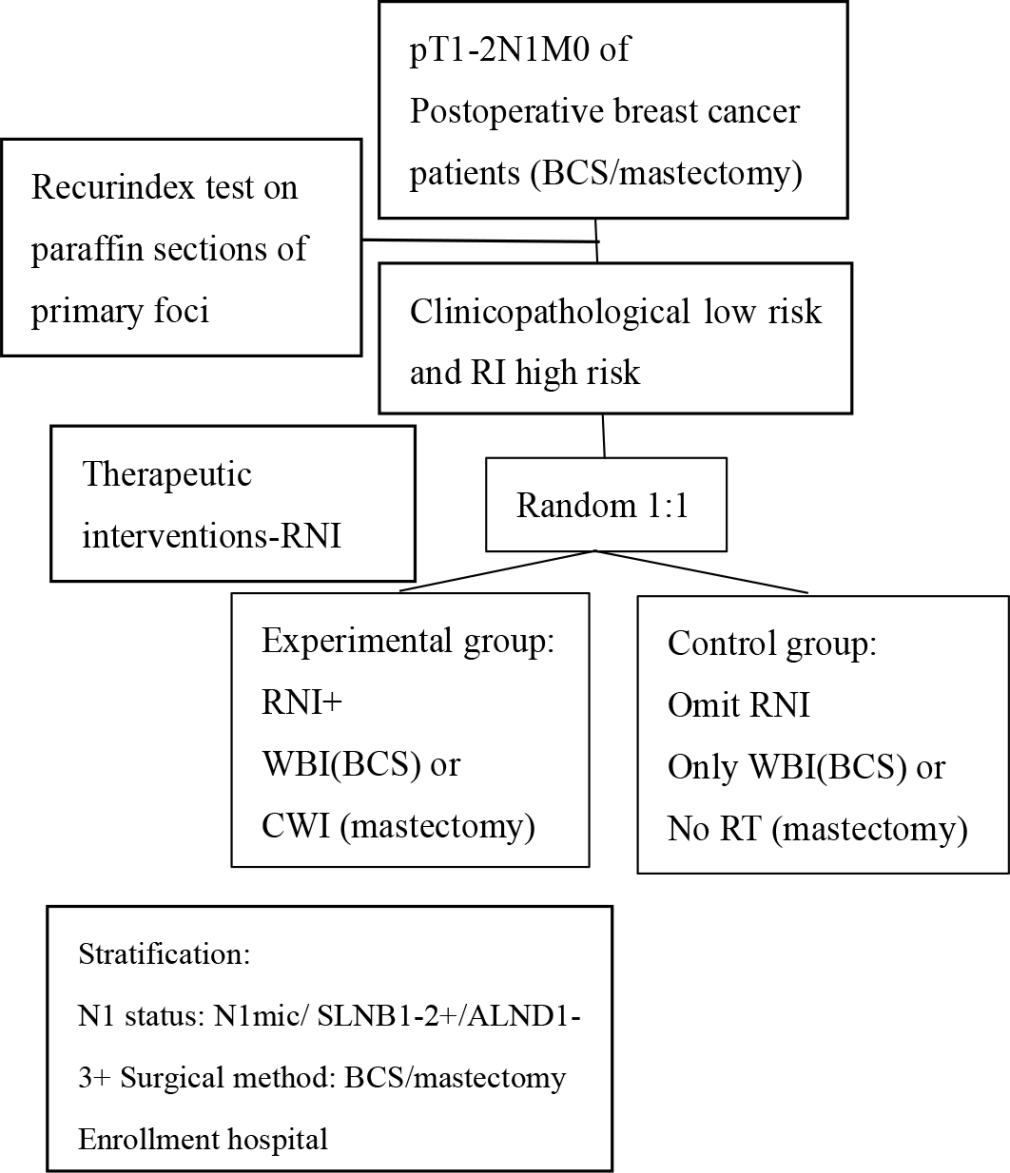
Research process. ALND, axillary lymph node dissection; BCS, breast-conserving surgery; CWI, chest wall irradiation; RI/RecurIndex, Recurrence Index; RNI, regional lymph node irradiation; RT, radiotherapy; SLNB, sentinel lymph node biopsy; WBI, whole breast irradiation.

The RIGAIN (RecurIndex-Guided postoperative radiotherapy with or without Avoidance of Irradiation of regional Nodes in 1–3 node-positive breast cancer) Study is a multicentre, prospective, randomised, open-label, phase III clinical trial that is being conducted in China. The overall research process is illustrated in figure 1. This study aims to screen postoperative patients with early-stage breast cancer (pT1–2N1M0) who have completed standard systemic therapy and possess eligible pathological specimens for participation. The inclusion and exclusion criteria are listed in box 1. RecurIndex testing will be performed using postoperative paraffin-embedded tissue sections from the primary lesion. The study is divided into a randomised controlled trial and an observational study based on clinical risk and RecurIndex-LRR risk. Patients with low clinical risk but high RecurIndex-LRR risk will be randomly assigned in a 1:1 ratio to either the experimental group (RNI) or the control group (no RNI), while patients with low clinical risk and low RecurIndex-LRR risk will be included in the observational study. This article primarily focuses on the randomised controlled trial. The study participants will receive the following treatments: experimental group: for patients who underwent BCS, RNI will be performed in combination with whole breast irradiation (WBI)+tumour bed boost irradiation. For patients who underwent mastectomy, chest wall irradiation (CWI) will be administered in combination with RNI. Control group: RNI will be omitted. For patients who underwent BCS, only WBI+tumour bed boost irradiation will be administered. For patients who underwent mastectomy, both RNI and CWI will be omitted. A comparative effectiveness analysis will be conducted. The study commenced in April 2023.

Box 1 **Eligibility criteria for the study**

**Inclusion criteria**

1. Age ≥18 years, ≤70 years.
2. Eastern Cooperative Oncology Group Performance Status ≤2 (online supplemental file 17).
3. Postoperative pathology confirms the diagnosis of invasive breast cancer.
4. Meets the clinical definition of low risk: (1) axillary lymph node micrometastasis (N1mic), or (2) N1 patients who meet all of the following conditions: (a) age ≥40 years; (b) lymphovascular invasion (LVI) negative or limited to individual or small foci of LVI (excluding extensive or large amounts of LVI); (c) three clinical molecular subtypes (luminal A type, luminal B1 type and luminal B2 type) are allowed in this study: oestrogen receptor (ER)-positive (ER ≥1%) and human epidermal growth factor receptor 2 (HER2)-negative or ER-positive (ER ≥1%) and HER2 overexpressing, respectively.
5. Postoperative pathological diagnosis of axillary lymph node status as any of the following: (a) sentinel lymph node biopsy or axillary lymph node dissection with micrometastasis (N1mic), (b) sentinel lymph node biopsy with one to two lymph node macrometastases (N1sln), (c) sentinel lymph node biopsy+axillary lymph node dissection or simple axillary lymph node dissection with one to three lymph node metastases (N1).
6. The primary tumour and breast underwent breast-conserving surgery or mastectomy±breast reconstruction (autologous/prosthetic).
7. A thorough systemic examination (eg, chest X-ray, ultrasound, CT, etc) within 3 months before randomisation for radiotherapy must confirm no distant metastasis.
8. Mammography and/or MRI within 12 months before surgery or randomisation for radiotherapy must confirm no contralateral breast cancer.
9. Postoperative completion of at least four cycles of adjuvant chemotherapy containing anthracycline or taxane regimens.
10. Radiotherapy must be performed sequentially after the completion of all adjuvant chemotherapy, starting no later than 8 weeks after the end of chemotherapy.
11. Patients must have sufficient postoperative paraffin tissue sections of the primary tumour for Recurrence Index testing.
12. No history of other malignant tumours, except for basal cell carcinoma of the skin.
13. Signed informed consent before the start of the study.

**Exclusion criteria**

1. Confirmed T3–4, N0, N2–3, M1 stage disease before postoperative radiotherapy enrolment.
2. Received any neoadjuvant treatment before surgery, including chemotherapy, endocrine therapy, targeted therapy or radiotherapy.
3. Patients who underwent mastectomy and only had sentinel lymph node biopsy.
4. History of contralateral breast cancer or other second primary malignant tumour (excluding basal cell carcinoma of the skin and cervical carcinoma in situ).
5. History of chest radiotherapy.
6. Presence of severe heart, lung, liver, kidney, haematopoietic system or nervous system diseases, or mental disorders.
7. Presence of scleroderma or active systemic lupus erythematosus or other autoimmune diseases.
8. Pregnant and breastfeeding patients.

### Randomisation method

Stratified randomisation will be used for the randomised study. For the active postoperative radiotherapy trial involving the clinically low LRR risk but high RecurIndex-LRR risk population, participants will be stratified by N1 status, surgical method and enrolling hospital, and then randomised in a 1:1 ratio into the experimental and control groups.

Randomisation stratification factors are as follows:

1. N1 status: N1mic or one to two lymph node macrometastases (including N1sln), or three lymph node macrometastases.
2. Surgical method: BCS or mastectomy.
3. Multicentre enrolling hospital.

A central randomisation system was developed by TaiMei Medical Technology Company to facilitate the randomisation process. Statistical experts responsible for randomisation designed the randomisation parameters in advance, allowing the system to generate a random allocation table. The main clinical trial centres conduct eligibility screening for potential participants. Once deemed eligible, the researchers at each subcentre access the server via the internet and enter the information of the enrolled patients. The system then assigns a corresponding randomisation number based on the random allocation table, determining the patient’s placement in the respective study group.

### Participants and recruitment

Patients will be recruited by radiation oncologists from each participating research centre. For each interested patient, the clinician or clinical coordinator will provide a complete and comprehensive introduction to them or their designated representative, informing the patient about their rights, the risks involved and the potential benefits they may receive to enhance their compliance with the protocol. Prior to enrolment, patients are required to sign an informed consent form, which will be kept in the case report form (CRF). Patient registration is scheduled to begin on 1 April 2023 (see online supplemental file 1 for details), and is expected to continue for 5 years (tentatively until January 2028). The final collection of data for the primary outcome measures is anticipated to be completed by December 2032.

### Patient and public involvement

Neither patients nor the public were involved in the design, conduct, reporting or dissemination plans of this study.

### Objectives and endpoints

This study aims to evaluate whether adjuvant radiotherapy to the regional lymph nodes after BCS or chest wall plus regional lymph node radiotherapy after total mastectomy can further improve clinical outcomes in N1 patients with low clinical risk but high RecurIndex-LRR risk.

The primary endpoint is invasive disease-free survival (IDFS). Secondary endpoints are AFR, LRR-free survival (LRFS), DMFS, RFS, OS, DFS, BCSM and patient quality of life assessment. The specific definitions can be found in table 1.

**Table 1:** The specific definitions of the study endpoints

IDFS	The time from the day the subject is randomised to the earliest occurrence of invasive cancer local recurrence, distant metastasis or death, but does not include contralateral breast second primary cancer.
AFR	Any ipsilateral chest wall, breast, regional lymph node recurrence or distant metastasis event that occurs during the follow-up period.
LRFS	The time from the day the subject is randomised to the earliest occurrence of ipsilateral chest wall, breast, or regional lymph node recurrence or death.
DMFS	The time from the day the subject is randomised to the earliest occurrence of distant metastasis or death.
RFS	The time from the day the subject is randomised to the earliest occurrence of ipsilateral chest wall, breast, regional lymph node recurrence, distant metastasis or death.
OS	The time from the day the subject is randomised until the patient’s death.
DFS	The time from the day the subject is randomised to the recurrence of the disease or the patient’s death due to disease progression.
BCSM	The time from the day the subject is randomised to death from breast cancer.

AFRany first recurrenceBCSMbreast cancer-specific mortalityDFSdisease-free survivalDMFSdistant metastasis-free survivalIDFSinvasive disease-free survivalLRFSlocal–regional recurrence-free survivalOSoverall survivalRFSrecurrence-free survival

During the screening period and 3 months after the end of treatment, patients in each group fill out the quality of life questionnaire (EORTC Core Quality of Life Questionnaire (EORTC QLQ-C30)), as well as the breast cancer survival quality scale (EORTC Breast Cancer-Specific Quality of Life Questionnaire (EORTC QLQ-BS23)) annually during the follow-up phase (onlinesupplemental files 2  3).

### RecurIndex test

RecurIndex testing was performed using postoperative paraffin-embedded tissue sections from the primary lesion of the subjects. All sections were uniformly sent to the Jiangsu Simcere Pharmaceutical Co, Jiangsu Simcere Diagnostics Co for free testing. Formalin-fixed, paraffin-embedded tissue blocks should be selected that cover the largest amount of tumour cells and meet the diagnostic criteria in appearance. Tissue sections with an excess amount of normal tissue, necrotic tissue, adipose tissue or haemorrhagic tissue should not be sent for examination. The tumour cell content in the identified sections should be >50% for the test to be performed. A total of 10 consecutive sections are needed, each with a thickness of 5 µm. The sections can remain unstained and without coverslips. There is no need to oven-dry the sections; they can be air-dried naturally.

### Safety assessment indicators

All patients participating in the RIGAIN Study are required to undergo safety assessments, including acute radiation reactions and late radiation injuries for radiotherapy patients. The evaluation criteria and handling of injuries are detailed in onlinesupplemental files 4^7^. Their treatment is shown in online supplemental file 8, including acute skin reactions to radiotherapy, symptomatic radiation pneumonitis, long-term cosmetic outcomes (BCS/reconstruction patients), skin fibrosis (total mastectomy patients), ischaemic heart disease, upper limb oedema,^27 28^ brachial plexus injury and second primary tumour.^29^

### Radiotherapy

#### General consideration

The overall treatment plan for each participant is determined by the researchers at the corresponding subcentre based on the participant’s condition. Depending on their assigned group, patients will either undergo RNI or be exempted from it. Breast-conserving patients will all receive WBI. Patients should start radiotherapy within 8 weeks after completion of adjuvant chemotherapy. The regional lymph nodes include the supraclavicular lymph nodes and infraclavicular lymph nodes (unresected levels II/III axillary lymph nodes), with or without internal mammary lymph nodes (at least from the first to the third intercostal space). For patients with minimally positive sentinel lymph node and without axillary lymph node dissection, the inclusion criteria encompass the low/intermediate axillary lymph nodes. The planned endocrine therapy and anti-human epidermal growth factor receptor 2 (HER2) treatment can be continued during the radiotherapy process.

#### Patient positioning and immobilisation

The patient lies on a fixed device such as a breast support, vacuum bag or foam pad. A CT scan is performed with a thickness of 3–5 mm, from the second cervical vertebra to the second lumbar vertebra. CT positioning includes surface marking, where lead wires are placed on the surgical scar of the primary lesion in breast-conserving patients or the chest wall scar in total mastectomy patients, as well as on the scar of the axillary sentinel/clearance lymph node incision. If there is a drainage site, it should also be separately marked with a lead wire or lead point.

#### Volumes of interest

The clinical target volume (CTV) and organs at risk (OARs) must be delineated on all CT slices, following the contouring guidelines of the Radiation Therapy Oncology Group (RTOG) and considering the actual situation at each CT slice. Detailed descriptions of CTV and OARs can be found in online supplemental file 9. The margin between the planning target volume (PTV) and CTV depends on the institutional standards of each participating centre, with a recommended minimum of 5 mm. Contours should be drawn according to the RTOG guidelines, including the ipsilateral and contralateral lungs, heart, humeral heads and spinal cord.

#### External beam equipment and techniques

Radiation therapy techniques that can be employed include three-dimensional conformal radiotherapy, forward intensity-modulated radiotherapy, inward intensity-modulated radiotherapy, volumetric modulated arc therapy and helical tomotherapy. Conventional radiotherapy (using a simulator for positioning and a two-dimensional planning system to design treatment plans with external and tangential fields) and proton therapy techniques are not allowed. Some variations in treatment planning and implementation are permitted to accommodate the participating centres in adapting to the research protocol. However, it is strongly recommended that the treatment plans for enrolled patients at each centre remain consistent to avoid confusion.

#### Dose prescription and fractionation

The whole breast target volume, or the integrated target volume of the whole breast and low to moderate axillary region, or the chest wall target volume, and the regional lymph node target volume receive a radiation dose of 5000 cGy in 25 fractions, delivered at a rate of 200 cGy per day, 5 days per week. Alternatively, a hypofractionated radiotherapy scheme can be chosen, with a radiation dose of 4000–4256 cGy in 15–16 fractions. For breast-conserving patients, a sequential tumour bed boost is performed after completion of WBI, as determined by individual centre investigators. It can be delivered using conventional fractionation, with a dose of 1000 cGy in 5 fractions at a rate of 200 cGy per day, or by using hypofractionation, with a dose of 798–1064 cGy in 3–4 fractions at a rate of 266 cGy per day. If there are high-risk factors for local recurrence, such as positive surgical margins, close margins or young age, the radiation dose for the tumour bed boost may be increased to 1400–1600 cGy in 7–8 fractions at a rate of 200 cGy per day (see online supplemental file 10 for details).

#### Dose and Volume Histogram (DVH) constraints

It is required that at least 95% of the prescribed dose to the PTV covers 95% of the PTV. The specific dose distribution is determined by each centre’s policy, with a recommended level as shown in online supplemental file 11. For breast-conserving patients, it is recommended achieving a central axis dose uniformity of ≤±7% for PTV_2 (whole breast or integrated target volume of whole breast and low to moderate axillary region) and PTV_1 (tumour bed), and minimising the volume receiving ≥105% of the prescribed dose. The constraints for OARs should follow the Quantitative Analysis of Normal Tissue Effects in Clinical guidelines (see online supplemental file 12).

### Withdrawal from research and study termination

#### Termination of treatment

Research treatment will be terminated if any of the following conditions occur in the patient:

1. The subject withdraws informed consent.
2. Any adverse event (AE) causes the subject to be unable to continue participating in the study.
3. The subject is lost to follow-up.
4. The subject does not comply with the study requirements and/or the investigator’s instructions.
5. The subject has a concomitant illness or change in the subject’s condition, and the investigator believes the subject is no longer suitable for the study treatment.
6. For any other reason the investigator believes the subject is not suitable for continuing in the study.

If a subject drops out or withdraws, relevant safety and efficacy evaluations should be completed as soon as possible.

#### Study termination

The trial will be terminated if any of the following situations occur during the trial:

1. Serious safety issues arise during the trial.
2. There is a major error in the study protocol.
3. The principal investigator voluntarily stops the trial.
4. The administrative authority revokes the trial.
5. The termination of the trial may be temporary or permanent.

If the trial is terminated, all trial records should be retained for review.

#### Follow-up evaluation and toxicity assessment

The registration timeline, intervention measures and assessments are presented in online supplemental file 13. In the follow-up phase after radiotherapy, check-ups and assessments will be performed every 6 months until the occurrence of an endpoint event or the end of the study. For patients without postoperative radiotherapy, check-ups and assessments will be conducted every 6 months after the completion of adjuvant chemotherapy until the occurrence of an endpoint event or the end of the study. Effectiveness evaluations include tumour imaging examinations and assessments, brain MRI or CT, bone scans, quality of life questionnaires (EORTC QLQ-C30) and breast cancer-specific quality of life scales (EORTC QLQ-BS23). Safety evaluations include but are not limited to physical examinations, Eastern Cooperative Oncology Group Performance Status (ECOG PS) scores, pregnancy test checks, blood routine tests, blood biochemistry tests, AEs and serious AEs (SAEs). At the end of the study, participants will undergo physical examinations, performance status assessments (ECOG PS), blood routine tests, blood biochemistry tests, tumour markers, breast ultrasound/MRI, mammography, chest X-ray/CT, abdominal ultrasound/CT, and EORTC QLQ-C30 and EORTC QLQ-BS23 scoring. Evaluations of concomitant medications and AEs are also required. The end-of-study visit window is 60 months after the last participant completes radiotherapy. For participants who are withdrawn or drop out before the end of the study, safety and effectiveness assessments will be conducted according to the requirements of end-of-study safety and effectiveness visits.

### Data management and quality assurance

In this study, electronic CRFs (eCRFs) are used to collect data, and the EDC system designated by the principal investigator is used to complete the eCRF. Monitors verify the original data to ensure that the data entered into the eCRF by authorised trial centre personnel (ie, original data) are accurate, complete and derived from original documents. Researchers and trial institutions must provide monitors with direct access to applicable source documents and reports for inspection and Independent Ethics Board/Ethics Committe review (see online supplemental file 14 for details). A Data Safety Committee has also been established, consisting of five members who are independent of the project team and have signed a research confidentiality agreement. The main tasks of the committee are to review and analyse positive results (recurrence and metastasis of subjects) and to understand the actual research results (without statistical analysis) when half of the subjects are enrolled. The committee will vote on whether it is necessary to adjust the research plan. This protocol does not include investigational drugs, and any toxicities that occur during treatment should be reported to the principal investigator and their Ethics Committee. In addition, the subcentres should also report to the Ethics Committee of their institution. All SAEs and other AEs must be recorded in the CRF. Furthermore, we have established a comprehensive quality control standard operating procedure, as detailed in online supplemental file 15.

### Sample size estimation

This study is designed for superiority, referring to authoritative postoperative radiotherapy studies MA20^10^ and EORTC 22922^11^ for N1 patients, in which the 5-year IDFS in the radiotherapy group and the control group was 90.7% vs 81.9% and 87.7% vs 77.1%, respectively. In the domestic RecurIndex external validation retrospective study^21^ for patients with N1 breast cancer, the 5-year IDFS in the high RecurIndex-LRR risk group was 81.1% vs 69.7% in the postoperative radiotherapy group and the control group, respectively. It is expected that the 5-year IDFS for the clinically low LRR risk and high RecurIndex-LRR risk population in the experimental group and control group in this study will be 89% and 82%, respectively. The superiority margin is set to improve the primary endpoint IDFS by ≥7% (HR=0.587) in the postoperative radiotherapy research group compared with the control group. With a one-sided significance level (α) of 0.025 and a power (1–β) of 0.8, assuming the experimental group performs better than the control group, the required sample size for each group was calculated as 216 cases per group using PASS V.15.0 software. The allocation ratio between the experimental and control groups was set at 1:1. Considering a 5-year enrolment period, 5-year follow-up period and potential 20% dropout rate (mainly considering the need for further 10-year and 15-year long-term efficacy follow-up after reaching the 5-year endpoint), each group will need 270 cases, totalling 540 cases.

### Statistical analysis

Descriptive statistical analyses will be conducted based on demographic characteristics such as age, gender, height, weight and other baseline characteristics such as medical history. The Cox proportional hazards model will be used for analysis of the primary endpoint. The HR and its 95% CI will be calculated, including stratification factors and other covariates. Additionally, the Cox proportional hazards model without covariates will be used to support the analysis results of the primary endpoint. Moreover, the Kaplan-Meier (KM) method will be used to calculate the median IDFS for the two groups (experimental group vs control group), including 95% CIs. KM plots will be used to illustrate the time trends of IDFS. For the secondary endpoints, the AFR of each group will be calculated, as well as the corresponding 95% Clopper-Pearson CIs. LRFS, DMFS, RFS, OS, DFS and BCSM will be analysed for median values using the KM method (including 95% CIs). The overall changes in EORTC QLQ-C30 and EORTC QLQ-BS23 scores from baseline will be summarised. Safety analysis will be conducted by summarising AEs, changes in laboratory test results, changes in vital signs and study treatment exposure. The results will be reported by treatment group. All AEs during treatment, grade 3 or higher treatment-emergent AEs (TEAEs), SAEs, radiotherapy-related SAEs and TEAEs leading to study termination will be summarised by organ system, preferred term, and group in terms of numbers and percentages (see online supplemental file 16 for details). A p value of ≤0.05 in a two-tailed test will be considered statistically significant. Statistical analyses will be performed using SPSS V.25.0 and STATA V.14.

### Ethics and dissemination

This study has obtained approval from the Ethics Committee of Sun Yat-Sen Memorial Hospital, Sun Yat-Sen University (SYSKY-2022-097-02, V.3.1), as well as approval from the respective participating centres’ Ethics Committees. The study is being conducted in accordance with the Helsinki Declaration and Good Clinical Practice. Approval from the Chinese Human Genetic Resources Office was obtained on 6 January 2021, with the reference number 2020SQCJ2358. The study was registered on ClinicalTrials.gov on 7 July 2022, with the registration number NCT04069884. The research findings will be published in peer-reviewed journals. The authors will be individuals who have made significant contributions to the study, design and implementation. Any modifications to the study protocol and informed consent documents must be reviewed and approved by the Ethics Committee before implementation.

### Confidentiality and protection of participants’ rights and interests

Researchers are required to explain to participants that participation in the clinical trial is voluntary, and that they have the right to withdraw from the study at any stage without affecting their medical treatment and rights. Personal information of participants will be kept confidential. Participants should be informed about the nature, purpose, potential benefits and possible risks of the clinical trial, as well as alternative treatment options. Researchers should ensure that the rights and obligations of participants, as stipulated in the declaration, are protected. Participants should be given adequate time to consider whether to participate and to sign the informed consent form.

## Discussion

The RIGAIN Study is a multicentre, open-label, randomised controlled, phase III clinical trial. Our objective is to precisely assess patients with clinically low LRR risk N1 breast cancer who, if identified as high risk for LRR by the RecurIndex test, may receive enhanced clinical efficacy from active RNI after BCS or CWI with RNI after mastectomy. The aim is to accurately identify patients who would benefit from intensified radiotherapy. In addition, we have established an observational study to investigate the potential to exclude RNI for patients who are clinically low LRR risk and are identified as low risk for LRR by the RecurIndex. The primary endpoint of the study is LRR, with the aim of identifying truly low-risk patients for whom radiotherapy can be safely omitted from planned treatment regimens. Similarly, the MA39 study defines a clinically and genetically low-risk LRR N1 population (age ≥40 years, luminal A type, Oncotype DX score <18) based on comprehensive clinical pathology, molecular subtype and a multigene model. The anticipated results from the MA39 study could potentially guide personalised RNI decisions for patients who are found to be both clinically and genetically low risk. However, this study also has limitations. First, the multigene models used in this study were developed to predict the risk of distant metastasis, and there may be inconsistencies between the occurrence of LRR and the risk of distant metastasis in clinical patients. Second, future research results are primarily intended to guide the omission of postoperative radiotherapy in clinically low-risk and genomically low-risk N1 patients. However, the significance of postoperative radiotherapy in patients with intersecting risks, particularly those who are clinically low risk but genomically high risk, remains unclear. Lastly, this study does not provide direct evidence for the application of Oncotype DX in guiding treatment decisions for Asian patients.

Traditionally, postoperative radiotherapy has been lauded for decreasing LRR and for helping to diminish the risk of distant metastases.^10 11^ This has led to long-term improvements in DFS and breast cancer-specific survival, providing the ultimate benefit to patients. Notably, this is partly attributed to the prevention of reseeding from recurrences. Another part is attributed to the radiation-induced abscopal killing effect, which refers to a series of immunological responses induced by local high-dose radiotherapy that culminate in the elimination of tumours distant from the irradiation site.^27^ In the context of postoperative radiotherapy for patients with regionally lymph node-positive breast cancer, the abscopal effect is most pronounced in patients classified as pN1, where the survival benefit is most conspicuous.^9^ Compared with mastectomy, BCS better preserves the immune microenvironment, thereby enhancing the transformation and activation of the immune response following postoperative radiotherapy. This is the primary reason for our selection of IDFS as the main endpoint in this study.

Oncotype DX and MammaPrint assays primarily assess the overall recurrence risk and mainly guide chemotherapy and endocrine treatment.^16 18 30^ Previous studies have indicated a high concordance in predicting the risk of distant metastases between the RecurIndex and the MammaPrint and Oncotype DX assays. However, some discrepancies exist in assessing the risk of LRR. The TAILORx Study indicated that the RecurIndex predictive model may identify patients at risk of LRR more accurately than the Oncotype DX.^16 31 32^

The RecurIndex predictive model stands out among various multigene prediction models in early-stage breast cancer with the following unique characteristics and advantages: (1) unlike other models that only assess overall recurrence risk and are more biased towards the risk of distant metastases, RecurIndex can independently assess both the risk of LRR and distant metastases, making it more suitable to guide precision radiotherapy; (2) RecurIndex demonstrates predictive efficacy in populations with HER2 overexpression and triple-negative breast cancer, potentially serving as a precise predictor of LRR risk in patients with these two types of N1mic tumours, which could help guide individualised radiotherapy decisions.

The study focuses on the RecurIndex risk prediction model with the aim of guiding postoperative individualised radiotherapy for patients with pT1–2N1M0 breast cancer. Particular attention is paid to the ‘clinically low LRR risk’ but ‘genetically high-risk’ population to explore and validate the clinical benefits of postoperative radiotherapy. The study design stands out for its clinical applicability and innovation as well as strict adherence to ethical and clinical practice standards. It effectively addresses the research gap in precise radiotherapy for N1 patients with overlapping risk profiles, both domestically and internationally. The study could potentially revolutionise the practice of postoperative radiotherapy by transitioning from a discretionary approach solely based on clinical and pathological information to an individualised optimisation guided by clinical genetic risk.

We anticipate that the RIGAIN Study will generate high-quality evidence, establishing a precise risk assessment framework to guide optimised radiotherapy decisions for patients with N1 breast cancer.

## supplementary material

10.1136/bmjopen-2023-078049 online supplemental file 1

10.1136/bmjopen-2023-078049 online supplemental file 2

10.1136/bmjopen-2023-078049 online supplemental file 3

10.1136/bmjopen-2023-078049 online supplemental file 4

10.1136/bmjopen-2023-078049 online supplemental file 5

10.1136/bmjopen-2023-078049 online supplemental file 6

10.1136/bmjopen-2023-078049 online supplemental file 7

10.1136/bmjopen-2023-078049 online supplemental file 8

10.1136/bmjopen-2023-078049 online supplemental file 9

10.1136/bmjopen-2023-078049 online supplemental file 10

10.1136/bmjopen-2023-078049 online supplemental file 11

10.1136/bmjopen-2023-078049 online supplemental file 12

10.1136/bmjopen-2023-078049 online supplemental file 13

10.1136/bmjopen-2023-078049 online supplemental file 14

10.1136/bmjopen-2023-078049 online supplemental file 15

10.1136/bmjopen-2023-078049 online supplemental file 16

10.1136/bmjopen-2023-078049 online supplemental file 17
