# Supplementary material for: RecurIndex-Guided postoperative radiotherapy with or without Avoidance of Irradiation of regional Nodes in 1–3 node-positive breast cancer (RIGAIN): a study protocol for a multicentre, open-label, randomised controlled prospective, phase III trial
Source: BMJ Open. 2024 Jul 30;14(7):e078049. doi: 10.1136/bmjopen-2023-078049 (PMC11293409; doi:10.1136/bmjopen-2023-078049)
Supplement: online supplemental file 1 [file bmjopen-14-7-s001.pdf]

Supplementary 1. Trial registration data

Data category	Information
Primary registry and trial identifying number	Line 44 page 4
Date of registration in primary registry	April 1. 2023
Source(s) of monetary or material support	Sun Yat-Sen Memorial Hospital, Sun Yat-Sen University
Primary sponsor	Sun Yat-Sen Memorial Hospital, Sun Yat-Sen University
Secondary sponsor(s)	the Jiangsu Simcere Pharmaceutical Co., Ltd., Jiangsu Simcere Diagnostics Co., Ltd
Contact for public queries	Xiaobo Huang, MD. [huangxbo@mail.sysu.edu.cn]
Contact for scientific queries	Xiaobo Huang, MD. Sun Yat-Sen Memorial Hospital, Sun Yat-Sen University, Guangzhou, Guangdong, China
Public title	**RIGAIN Study**
Scientific title	Line 3 page 3
Countries of recruitment	Line 29 page 4
Health condition(s) or problem(s) studied	Regional lymph node irradiation
Intervention(s)	Line 26 page 4
Key inclusion and exclusion criteria	*Table 1* Line 3 page 18
Study type	Line 27 page 4
Date of first enrolment	Line 38 page 4
Target sample size	Line 33 page 4
Recruitment status	Recruiting
Primary outcome(s)	Line 19 page 8
Key secondary outcomes	Line 19 page 8
