# Supplementary material for: RecurIndex-Guided postoperative radiotherapy with or without Avoidance of Irradiation of regional Nodes in 1–3 node-positive breast cancer (RIGAIN): a study protocol for a multicentre, open-label, randomised controlled prospective, phase III trial
Source: BMJ Open. 2024 Jul 30;14(7):e078049. doi: 10.1136/bmjopen-2023-078049 (PMC11293409; doi:10.1136/bmjopen-2023-078049)
Supplement: online supplemental file 2 [file bmjopen-14-7-s002.pdf]

Supplementary 2. Quality of Life Questionnaire EORTC QLQ-C30 (version 3)

We are interested in learning some information about you and your health status. Please answer all of the following questions independently and circle the answer that is most appropriate for you. There are no "correct" or "incorrect" answers. The information you provide will be kept strictly confidential.

Please fill in your last name: ______________________

Date of birth (year, month, day): ______________________

Today's date (year, month, day): ______________________

	No	A little	Some	Very much
1.Do you feel difficulty when you do some laborious movements, such as lifting heavy shopping bags or luggage?	1	2	3	4
2. Do you find it difficult to walk long distances?	1	2	3	4
3. Do you find it difficult to walk short distances outdoors?	1	2	3	4
4. During the day, do you have to lie in bed or sit in a chair?	1	2	3	4
5. Do you need assistance with eating, dressing, washing or going to the bathroom?	1	2	3	4
In the past week:	1	2	3	4
6. Are your work or daily activities limited by physical ability?	1	2	3	4
7. Are your hobbies and leisure activities physically limited?	1	2	3	4
8. Do you ever feel short of breath?	1	2	3	4
9. Have you ever had any pain?	1	2	3	4
10. Have you ever needed rest?	1	2	3	4
11. Have you ever felt sleep deprived?	1	2	3	4
12. Have you ever felt weak?	1	2	3	4
13. Have you ever felt a lack of appetite?	1	2	3	4
14. Have you ever felt nauseous and wanted to vomit?	1	2	3	4
15. Have you ever vomited?	1	2	3	4
16. Have you ever had constipation?	1	2	3	4
17. Have you ever had diarrhea?	1	2	3	4
18. Do you ever feel tired?	1	2	3	4
19. Does pain interfere with your daily activities?	1	2	3	4
20. Do you have difficulty concentrating on things, such as reading the newspaper or watching TV?	1	2	3	4
21. Do you ever feel nervous?	1	2	3	4
22. Do you ever feel worried?	1	2	3	4
23. Do you ever feel easily irritated?	1	2	3	4
24. Do you ever feel depressed?	1	2	3	4
25. Do you ever have trouble remembering things?	1	2	3	4
26. Has your medical condition or treatment process interfered with your family life?	1	2	3	4
27. Has your medical condition or treatment interfered with your social activities?	1	2	3	4
28. Has your medical condition or treatment process caused you financial difficulties?	1	2	3	4

For the following questions, the numbers 1-7 represent a scale from "very poor" to "very good".

29. How would you rate your overall health in the past week?

1	2	3	4	5	6	7
very poor" to						very good

30. How would you rate the overall quality of your life in the past week?

1	2	3	4	5	6	7
very poor" to						very good

Patients sometimes have the following clinical symptoms. Please indicate the extent of these clinical symptoms or problems you have had in the past week, circling the answer that best applies to you.

	No	A little	Some	Very much
31. Do you cough a lot?	1	2	3	4
32. Do you cough up blood (blood in sputum)?	1	2	3	4
33. Do you feel short of breath when you rest?	1	2	3	4
34. Do you feel short of breath when you take a walk?	1	2	3	4
35. Do you feel short of breath when climbing stairs?	1	2	3	4
36. Have you ever had pain in your mouth or tongue?	1	2	3	4
37. Have you ever had difficulty swallowing?	1	2	3	4
38. Have you ever had tingling/numbness in your hands and feet?	1	2	3	4
39. Have you ever had hair loss?	1	2	3	4
40. Have you ever had chest pains?	1	2	3	4
41. Have you ever had pain in your arms or shoulders?	1	2	3	4
42. Have you ever had any pain in other parts of your body?	1	2	3	4
If yes, please write down the area:				
43. Have you ever taken any painkillers?				
1.Yes 2.No				
If you have used it, does it help much with pain?	1	2	3	4
