# Supplementary material for: RecurIndex-Guided postoperative radiotherapy with or without Avoidance of Irradiation of regional Nodes in 1–3 node-positive breast cancer (RIGAIN): a study protocol for a multicentre, open-label, randomised controlled prospective, phase III trial
Source: BMJ Open. 2024 Jul 30;14(7):e078049. doi: 10.1136/bmjopen-2023-078049 (PMC11293409; doi:10.1136/bmjopen-2023-078049)
Supplement: online supplemental file 3 [file bmjopen-14-7-s003.pdf]

Supplementary 3. Breast Cancer Survival Quality Scale EORTC QLQ-BR23

Please recall if you have experienced any of these symptoms or the extent of the problem and tick the appropriate number"√"

In the past 1 week	No	A little	More	A lot
1. Do you have dry mouth?	1	2	3	4
2. Do your food and drinks taste different than usual?	1	2	3	4
3. Do your eyes hurt, feel uncomfortable, or tear up?	1	2	3	4
4. Do you have hair loss?	1	2	3	4
5. If you have hair loss, does it bother you?	1	2	3	4
6. Do you feel sick or uncomfortable?	1	2	3	4
7. Is your face red and hot?	1	2	3	4
8. Do you have a headache?	1	2	3	4
9. Do you feel less physically attractive due to illness or treatment?	1	2	3	4
10. Do you feel less attractive as a woman due to illness or treatment?	1	2	3	4
11. Do you have difficulty looking at your naked body?	1	2	3	4
12. Are you dissatisfied with your body?	1	2	3	4
13. Are you worried about your future health?	1	2	3	4

In the past 4 week				
14. How interested are you in sex?	1	2	3	4
15. How active are you sexually (do you have sex often)? (With or without sex?)	1	2	3	4
16. If you have sex, to what extent does it bring you pleasure?	1	2	3	4

In the past 1 week
17. Do you have pain in your arm or shoulder?
18. Is your arm or hand swollen?
19. Do you have difficulty lifting or moving your arm to the side?
20. Do you have pain in the area of your affected breast?
21. Is the area of your affected breast swollen?
22. Do you have hypersensitivity in the affected breast area?
23.Do you have skin problems (e.g. itching, dryness, flaking) in the affected breast area?
