# Supplementary material for: RecurIndex-Guided postoperative radiotherapy with or without Avoidance of Irradiation of regional Nodes in 1–3 node-positive breast cancer (RIGAIN): a study protocol for a multicentre, open-label, randomised controlled prospective, phase III trial
Source: BMJ Open. 2024 Jul 30;14(7):e078049. doi: 10.1136/bmjopen-2023-078049 (PMC11293409; doi:10.1136/bmjopen-2023-078049)
Supplement: online supplemental file 4 [file bmjopen-14-7-s004.pdf]

Supplementary 4. Evaluation criteria for common adverse events (CTCAE Version 4.03)

(excerpt, normal common adverse event evaluation criteria is grade 0)

Adverse Events	Grading				
	1	2	3	4	5
Hemoglobin g/L	Normal value -10.0	10. 0−8. 0	8. 0−6. 5	＜6. 5	
Leukocytes(10^9/L)	Normal value-3.0	3. 0−2. 0	2. 0−1. 0	＜1. 0	
Neutrophils(10^9/L)	Normal value-1.5	1. 5−1. 0	1. 0−0. 5	＜0. 5	
Platelets(10^9/L)	Normal value-75	75−50	50−25	＜25	
Transaminase ALT/AST	≤2. 5×N	2. 6−5. 0×N	5. 1−20×N	＞20×N	
Alkaline phosphatase	≤2. 5×N	2. 6−5. 0×N	5. 1−20×N	＞20×N	
Bilirubin	ULN−1. 5×N	1. 5−3. 0×N	3. 0−10×N	＞10×N	
Creatinine Cr	ULN−1. 5×N	1. 5−3. 0×N	3. 0−10×N	＞10×N	
Weight gain/loss	5. 0−10%	10−20%	≥20%		
Vomiting	Vomiting 1 time in 24h during treatment	Vomiting 2-5 times in 24h during treatment	Vomiting ≥ 6 times in 24h during treatment or requiring fluids	Life-threatening and requires urgent treatment	Death
Coughing sputum	Occasional/mild coughing of sputum	Moderate cough and sputum; interferes with instrumental daily life	Persistent heavy coughing and limited personal self-care		
Pneumonia	Asymptomatic; clinical examination or diagnostic findings only; no intervention required	Symptomatic (mild cough and/or dyspnea, with or without fever); requires clinical intervention; interferes with instrumental daily	Severe symptoms; limited personal autonomy; need for oxygen	Life-threatening respiratory dysfunction; requiring urgent treatment (tracheotomy or intubation)	Death

		life			
Acute coronary syndrome		Symptomatic, progressive angina; normal cardiac enzymes; hemodynamically stable	Symptomatic, unstable angina with/ or acute myocardial infarction, abnormal cardiac enzymatic parameters, hemodynamically stable	Symptomatic, unstable angina with/ or acute myocardial infarction, abnormal cardiac enzymatic parameters, hemodynamic instability	Death
Left ventricular systolic insufficiency			Symptoms of decreased ejection fraction	Uncontrollable heart failure with declining ejection fraction requiring urgent intervention	Death
Heart Failure	Asymptomatic, with abnormalities detected by laboratory tests (e.g., natriuretic peptide) or cardiac imaging	Mild to moderate symptoms with activity or exercise	Symptoms occur at rest or with light activity or exercise; requires treatment	Life-threatening; requires urgent treatment (e.g. continuous infusion therapy or mechanically assisted circulation)	Death
Limb edema	Comparison using the greatest difference in volume or circumference, with 5% to 10% variation between limbs; edema or blurred anatomy that can only be detected on close examination	Comparison using the largest difference in volume or circumference, 10% <~30% difference between limbs; disappearance of skin folds; apparent loss of limb anatomy, change in	>30% volume variation between limbs; severe changes in limb shape; limited personal autonomy		

		shape; interferes with instrumental daily living			
Neurotoxicity - Sensory	Mild sensory abnormalities (including paresthesia), absence of deep tendon reflexes	Moderate objective sensory deficit or sensory abnormalities (including tingling)	Severe objective sensory loss or sensory abnormalities that affect daily life	Persistent sensory loss, affecting function	Death
Neurotoxicity-motor	Self-perceived weakness with no objective findings	Moderate self-conscious weakness; no significant functional impairment	Self-perceived weakness with functional impairment	Paralysis	Death

ULN, Upper limit of normal value
