# Supplementary material for: RecurIndex-Guided postoperative radiotherapy with or without Avoidance of Irradiation of regional Nodes in 1–3 node-positive breast cancer (RIGAIN): a study protocol for a multicentre, open-label, randomised controlled prospective, phase III trial
Source: BMJ Open. 2024 Jul 30;14(7):e078049. doi: 10.1136/bmjopen-2023-078049 (PMC11293409; doi:10.1136/bmjopen-2023-078049)
Supplement: online supplemental file 5 [file bmjopen-14-7-s005.pdf]

Supplementary 5. Scoring criteria for late radiation injury (RTOG/EORTC 1995)

Organ Tissue	Grading				
	0	1	2	3	4
Skin	No change	Mild atrophy, hyperpigmentation, partial hair loss	Lamellar atrophy, moderate capillary dilatation, total hair loss	Significant atrophy, marked capillary dilation	Ulcers
Subcutaneous tissue	No change	Mild sclerosis (fibrosis) and loss of subcutaneous adipose tissue	Moderate fibrosis but asymptomatic, slight constriction of irradiated field <10% of the side length	Severe sclerosis and loss of subcutaneous tissue. Constriction of the irradiated field >10% border length	Necrosis
Lungs	No change	Asymptomatic or mildly symptomatic (dry cough), mild imaging signs	Moderate symptomatic pulmonary fibrosis or pneumonia (severe cough), hypothermia, patchy imaging	Severe symptomatic pulmonary fibrosis or pneumonia with dense imaging changes	severe respiratory insufficiency requiring continuous oxygenation or assisted ventilation
Heart	No change	Asymptomatic or mildly symptomatic; temporary T-wave inversion and ST changes; sinus tachycardia >110 beats/min at rest	Moderate exertional angina; mild pericarditis; normal heart size; persistent T-wave abnormalities and ST changes; low QRS waves	Severe angina pectoris; pericardial effusion; constrictive pericarditis; moderate heart failure; cardiac enlargement; abnormal electrocardiogram	pericardial tamponade; severe heart failure; severe constrictive pericarditis
