# Supplementary material for: RecurIndex-Guided postoperative radiotherapy with or without Avoidance of Irradiation of regional Nodes in 1–3 node-positive breast cancer (RIGAIN): a study protocol for a multicentre, open-label, randomised controlled prospective, phase III trial
Source: BMJ Open. 2024 Jul 30;14(7):e078049. doi: 10.1136/bmjopen-2023-078049 (PMC11293409; doi:10.1136/bmjopen-2023-078049)
Supplement: online supplemental file 6 [file bmjopen-14-7-s006.pdf]

Supplementary 6. Harris Cosmetic Grade Rating of Cosmetic Breast Preservation/Reconstructive Surgery Results

Level	Double breast symmetry	Double nipple level gap	Breast shape on the affected side	Skin
Excellent, Good	Symmetries	≤2cm	No significant difference with the healthy side, normal appearance, no deformation of the breast lift due to scarring, no difference between the affected side and the healthy side in feel	Normal
General	Symmetries	2cm-3cm	The shape of the affected breast is basically normal or slightly smaller than the healthy side, and the feel of the affected side is slightly worse.	Lightened or shiny color
Bad	Obvious asymmetry	＞3cm	The appearance of the affected side of the breast changes and is significantly smaller than the healthy side, and feels poorly in the hand	Thick, rubber-like, rough
