# Supplementary material for: RecurIndex-Guided postoperative radiotherapy with or without Avoidance of Irradiation of regional Nodes in 1–3 node-positive breast cancer (RIGAIN): a study protocol for a multicentre, open-label, randomised controlled prospective, phase III trial
Source: BMJ Open. 2024 Jul 30;14(7):e078049. doi: 10.1136/bmjopen-2023-078049 (PMC11293409; doi:10.1136/bmjopen-2023-078049)
Supplement: online supplemental file 7 [file bmjopen-14-7-s007.pdf]

Supplementary 7. Baker's classification of the prosthetic envelope

Grading	Breast Implants
I (no accessible envelope)	Breast implants feel as soft as non-operated breasts
II(Lightly hardened)	The softness of the breast is slightly worse, the implant can be touched but not seen
III(Heavy hardening)	Harder breasts, implants can be easily touched out or visible deformation of the implant
IV(severe contracture)	Breasts are hard, painful when touched, skin temperature becomes cold, deformation is obvious

Only Baker grade III and IV are defined as periosteal contracture and require reoperation
