# Supplementary material for: RecurIndex-Guided postoperative radiotherapy with or without Avoidance of Irradiation of regional Nodes in 1–3 node-positive breast cancer (RIGAIN): a study protocol for a multicentre, open-label, randomised controlled prospective, phase III trial
Source: BMJ Open. 2024 Jul 30;14(7):e078049. doi: 10.1136/bmjopen-2023-078049 (PMC11293409; doi:10.1136/bmjopen-2023-078049)
Supplement: online supplemental file 8 [file bmjopen-14-7-s008.pdf]

Supplementary 8. Radiotherapy-related adverse reactions and their treatment

1 Radiation-induced skin damage

Early skin reactions are those that occur within three months after the start of radiotherapy and are the most common complications in breast radiotherapy. Approximately 92% of patients receiving post-lumpectomy radiotherapy will experience acute radiation-induced skin reactions, mostly grade 1 or 2 mild reactions, with a wet desquamation incidence rate of about 3%. Patients undergoing mastectomy will almost always experience acute radiation-induced skin reactions, mostly grade 2 reactions, with a wet desquamation incidence rate of about 10-20%.

Prevention is the main approach to managing radiation-induced skin complications. For grade 1 and 2 injuries, conservative treatment is primarily used. Patients should wear loose, cotton open-front underwear, avoid friction and pressure on the skin in the irradiation area, avoid using irritating products such as soap and shower gel, avoid bathing with hot water or showering the irradiation area, and not apply chemical ointments or adhesive tape. If the skin is red, swollen, itchy, or painful, do not scratch it or apply medication randomly. Follow the doctor's advice for medication, such as triethanolamine cream, compound vitamin B12 solution, and medical radiation protectants. Wet dermatitis can be treated with exposure therapy, keeping the area dry and avoiding secondary infections. Wet dermatitis that does not heal after more than two months may develop into skin necrosis, often requiring surgical treatment, with skin grafting for larger areas.

Late skin reactions include local hyperpigmentation, telangiectasia, atrophy, and fibrosis. For chronic radiation dermatitis with recurrent ulceration and significant worsening, surgery is often used to prevent malignant transformation.

2 Pharyngeal and esophageal reactions

Irradiation of the supraclavicular area can cause pharyngeal pain and difficulty swallowing, which are generally mild and self-limiting. Prevention methods include using new radiotherapy techniques, accurate positioning, precise delineation of the target area, rational design of radiation fields, and reducing or avoiding irradiation of organs at risk. Symptomatic support treatment is provided for severe reactions.

3 Radiation-induced lung injury

Radiation-induced lung injury includes early radiation pneumonitis occurring within 3 months after radiotherapy and late radiation-induced pulmonary fibrosis occurring after 3 months. Approximately 2/3 of patients will develop asymptomatic radiation pneumonitis, which does not require treatment. The incidence of symptomatic radiation pneumonitis is between 1% and 5%, usually occurring within 2 months after radiotherapy or within 6 months after radiotherapy. Patients have symptoms and signs of pneumonia, which can manifest as cough, sputum, or fever, and in severe cases, dyspnea and hypoxia. In particular, when imaging examinations (chest X-rays and CT scans) show inflammatory exudative changes in the lung tissue within the irradiation field, symptomatic radiation pneumonitis can be diagnosed after excluding lung metastasis and tuberculosis. Supportive treatment, including hormones, oxygen therapy, and even mechanical ventilation, can provide complete relief, but some patients may still develop pulmonary fibrosis within 6-12 months, even with treatment. Pulmonary fibrosis is a late injury caused by damage to the lung interstitium and pleura, and in severe cases, it can be life-threatening.

There is currently no specific treatment for radiation pneumonitis, so prevention is more important than treatment. For patients undergoing whole-breast irradiation alone, it is recommended to use a dose-volume constraint of V20 < 22% for the ipsilateral lung. For those receiving irradiation of the supraclavicular lymph node region, a dose-volume constraint of V20 < 34% and V30 < 22% should be used for the ipsilateral lung to further evaluate the overall radiotherapy plan.

4 Radiation-induced heart damage

Radiation-induced heart disease (RIHD) initially manifests as acute pericarditis and later as coronary artery disease, chronic pericarditis, myocardial fibrosis, cardiomyopathy, heart valve damage, and cardiac conduction abnormalities. A 2013 New England Journal article reported that for every 1 Gy increase in the average dose to the heart, the incidence of major coronary events increased by 7.4%.

Reducing the risk of RIHD is also focused on prevention. The most fundamental measure is to minimize or avoid radiation exposure to the heart during radiotherapy. The Chinese Anti-Cancer Association Breast Cancer Diagnosis and Treatment Guidelines and Standards (2015 Edition) recommend that the average radiation dose to the heart should be assessed to be at least below 8 Gy. It is recommended to limit the heart's V30 to less than 10%. In addition, for high-risk populations of RIHD or those with cardiovascular disease, drugs that have a protective effect on the cardiovascular system should be used as soon as possible. Regular cardiac ultrasound follow-ups should be conducted during the follow-up phase.

5 Upper limb edema

Edema in the affected upper limb is one of the common complications after breast cancer surgery and/or radiotherapy, and the extent of surgery is an important influencing factor. AMAROS research reported that the 1-year, 3-year, and 5-year lymphedema incidence rates for the ALND group were 28%, 23%, and 23%, respectively, significantly higher than the 15%, 14%, and 11% for the SLNB + axillary radiotherapy group. The incidence of upper limb lymphedema after axillary lymph node biopsy alone is 5%. Edema caused by radiotherapy usually occurs 1 to 2 months after the end of radiotherapy. Depending on the time of onset, upper limb edema can be divided into early edema and delayed edema occurring after several years. Upper limb edema caused by tumor recurrence in the axilla and supraclavicular region is not considered a true post-treatment complication.

The main prevention method for upper limb edema is to reduce axillary dissection, and postoperative progressive functional exercise is the key to preventing upper limb edema. When upper limb edema occurs, manual massage or compression therapy can be used.

6 Brachial plexus nerve injury

Radiation-induced brachial plexus nerve injury is a rare late complication after breast cancer radiotherapy, with an incidence rate of 1%-4%. Early symptoms include sensory and motor disorders in the affected limb and pain, often accompanied by severe nocturnal pain. Some cases may also have lymphedema, with progressively worsening functional impairment. In the late stage, this can lead to the loss of function of the entire limb, causing lifelong disability for the patient and severely affecting the patient's daily life and rest, with a significant impact on their mental health and quality of life. A preliminary diagnosis can be made based on the patient's radiotherapy history, asymptomatic intervals, and clinical features in clinical practice. However, it is necessary to rule out brachial plexus nerve injury caused by tumor metastasis or compression.

Radiation-induced brachial plexus nerve injury is irreversible, and there is currently no ideal treatment

method, so prevention is crucial. It is essential to strictly follow the indications for radiotherapy in the lymphatic drainage area and pay attention to the radiotherapy range and radiation dose. For cases without severe pain, active measures should be taken to improve the blood supply of the nerves and surrounding soft tissues, and the earlier the diagnosis and treatment, the better the results. For advanced cases, treatment is aimed at relieving pain and improving quality of life.

7 Second primary tumors

Second primary tumors that can occur after breast cancer treatment include contralateral breast cancer and other malignant tumors such as lung cancer and soft tissue sarcomas. If these second primary tumors can be diagnosed and treated early, they do not affect the patient's survival. Therefore, regular follow-up of patients should be strengthened in clinical practice.

8 Rib fractures

The incidence is less than 1%. In most cases, patients have no noticeable symptoms, and fractures are discovered during bone scans or X-ray examinations. A small number of patients may experience chest wall or rib pain, which generally heals on its own without the need for special treatment.

9 Other side effects

During radiotherapy, patients may experience mild loss of appetite and fatigue. Therefore, it is important to adjust the diet reasonably, advocating for a "high protein, high vitamin, low fat" diet to maintain a balanced nutrition. Regularly review routine blood tests, and if a decrease in white blood cells is found, there is a risk of infection. In such cases, it may be necessary to temporarily pause radiotherapy and follow the doctor's advice for symptomatic supportive treatment.
