# Supplementary material for: RecurIndex-Guided postoperative radiotherapy with or without Avoidance of Irradiation of regional Nodes in 1–3 node-positive breast cancer (RIGAIN): a study protocol for a multicentre, open-label, randomised controlled prospective, phase III trial
Source: BMJ Open. 2024 Jul 30;14(7):e078049. doi: 10.1136/bmjopen-2023-078049 (PMC11293409; doi:10.1136/bmjopen-2023-078049)
Supplement: online supplemental file 9 [file bmjopen-14-7-s009.pdf]

Supplementary 9. Radiation Target Volume Naming and Delineation

I. Purpose:

To ensure the smooth conduct of the clinical trial and to guarantee the quality of the clinical trial.

II. Scope:

This clinical trial.

III. Procedures:

一、General Principles of Target Delineation: To be performed on plain CT scans.

二、RNI + WBI (BCS)/CWI (Mastectomy)

**Standards**	**2.1 Whole Breast Target CTV_2**
Superior Boundary	Upper edge of the palpable/CT-visible gland.
Inferior	Lower edge of the palpable/CT-visible gland.
Anterior	5 mm beneath the skin; for small and thin breasts, adjust the anterior boundary to 0.3 cm beneath the skin or even closer.
Posterior	1-2 mm behind the surface of the pectoralis major fascia (adjacent to the retromammary space), leaving no fat gap, including the lymph nodes between the pectoralis major and minor muscles and unsampled axillary levels I and II, excluding ribs/intercostal muscles.
Medial	Parasternum, at least to the medial edge of the internal mammary vessels.
Lateral	Lateral edge of the palpable/CT-visible gland, anterior to the thoracodorsal artery, and anterior edge of the latissimus dorsi muscle.

**Standards**	**2.2Tumor Bed and CTV_1**
Tumor Bed	The boundaries of the tumor bed are determined by: The positions of the surgical clips; it is recommended to place clips at five points: left, right, superior, inferior, and posterior.The extent of seroma, ensuring that any seroma within the gland and beneath the scar is included.
CTV_1	Includes the breast glandular tissue and soft tissue extending 10-15 mm beyond the surgical tumor bed. For patients who underwent segmental resection, a smaller margin of around 10 mm is recommended. If there is no glandular tissue beyond the tumor bed, the margin can be appropriately reduced. For patients with positive margins, extensive intraductal component (EIC), or severe atypical ductal hyperplasia (ADH), the margin must be appropriately expanded.

Standards	2.3 Integrated Target Volume CTV_2 for Whole Breast and Low/Mid Axillary Regions		
Whole Breast	Refer to the Whole Breast Target CTV_2		
Axilla	Axillary Level I: Anatomically marked by the lateral edge of the pectoralis minor.		
	Axillary Level I	Axillary Level II	Rotter’s Lymph Nodes
Superior	Where the axillary vessels cross the lateral edge of the pectoralis minor	Where the axillary vessels cross the medial edge of the pectoralis minor	Includes the superior side of the axillary artery and 5 mm above the axillary vein
Inferior	Where the pectoralis major inserts into the ribs	Where the axillary vessels cross the lateral edge of the pectoralis minor	Inferior boundary of Axillary Level II
Anterior	Anterior surface of the pectoralis major and latissimus dorsi	Anterior surface of the pectoralis minor	Posterior surface of the pectoralis major
Posterior	Anterior surface of the subscapularis muscle	Ribs and intercostal muscles	Anterior surface of the pectoralis minor
Medial	Lateral edge of the pectoralis minor	Medial edge of the pectoralis minor	Medial edge of the pectoralis minor
Lateral	Medial surface of the latissimus dorsi	Lateral edge of the pectoralis minor	Lateral edge of the pectoralis minor

Standards	Chest Wall Target CTV_CW
Superior	Clinical markers/subclavian head 0.5-1 cm
Inferior	Clinical markers/inferior edge of the contralateral breast fold
Anterior	Skin, excluding the wire
Posterior	Ribs and intercostal muscles
Medial	Clinical markers/junction of the sternum and ribs
Lateral	Clinical markers/thoracodorsal vessels and the anterior edge of the latissimus dorsi muscle
**Note:**<br>1. The entire scar should be included, and the target area should not be reduced within 2 cm above and below the scar.<br>2. Postoperative changes visible on CT (such as granulomas, fibrosis, and spiculated muscle	

irritation signs) should be included.

**Standards**	**2.5 Supraclavicular and Infraclavicular Lymph Node Area CTV_LN**
Superior	Inferior edge of the cricoid cartilage
Inferior	0.5-1 cm below the clavicular head, at the level where the brachiocephalic vein disappears, merging with the whole breast/chest wall target area
Anterior	Superior part: posterior surface of the sternocleidomastoid muscle; Inferior part: posterior surface of the pectoralis major muscle
Posterior	Superior part: posterior edge of the anterior scalene muscle; Inferior part: anterior edge of the ribs and intercostal muscles
Medial	Superior part: internal jugular vein, covering the interscalene triangle to the level of the transverse cervical artery and vein; Inferior part: junction of the subclavian vein and internal jugular vein
Lateral	Superior part: lateral edge of the sternocleidomastoid muscle; Inferior part: lateral edge of the pectoralis minor muscle
**Note:**<br>1. Avoid the surgically treated axillary area (Level I and part of Level II).<br>2. Include the non-surgically treated area of axillary Level II.	

**Standards**	**2.6 Internal Mammary Lymph Node Area CTV_IMN**
Superior	Injection into the internal area of the clavicle; for high-risk patients, extend to the junction of the internal jugular vein, subclavian vein, or brachiocephalic vein, and the internal mammary vein
Inferior	Upper edge of the fourth rib cartilage
Anterior	Posterior surface of the pectoralis major muscle and the posterior surface of the sternum
Posterior	Pleura or 5 mm behind the posterior aspect of the internal mammary vessels
Medial	5 mm inside the internal mammary vessels, covering the space between the sternum and the vessels
Lateral	5 mm outside the internal mammary vessels, to the outer edge of the brachiocephalic vein
**Note:**<br>1. For high-risk patients, the superior boundary extends to the junction of the internal jugular vein, subclavian vein, or brachiocephalic vein, and the internal mammary vein.<br>2. It is recommended to extend the coverage in the medial and lateral directions (at least) by 5 mm.	

Standards	2.7 Intraclavicular Lymph Node CTV_intraclavicular-LN
Superior	Level of the transverse cervical artery
Inferior	Upper edge of the brachiocephalic trunk
Medial	Midline of the body
Lateral	Inner boundary of the upper clavicle region
**Note:**<br>1. When irradiating the internal mammary lymph nodes, routine delineation is recommended.<br>2. When there is capsular invasion of the lymph nodes in the axillary Level II/III region, routine delineation is recommended.<br>3. Patients with primary tumor invasion of the deep fascia or tumors located medially and superiorly within the breast may be considered for delineation.	

三、Omission of RNI, WBI (BCS) only, no CWI (total mastectomy)

Standard	3.1 Whole Breast Target CTV_2
Superior	Upper edge of palpable/CT-visible gland.
Inferior	Lower edge of palpable/CT-visible gland.
Anterior	Subcutaneous tissue 5 mm beneath the skin; for thin/small breasts, adjust anterior boundary to 0.3 cm beneath the skin or even closer.
Posterior	1-2 mm behind the surface of the pectoralis major fascia (adjacent to the retromammary space), leaving no fat gap, excluding lymph nodes between pectoralis major and minor muscles and unsampled axillary levels I and II, excluding ribs/intercostal muscles.
Medial	Parasternal, at least to the medial edge of the internal mammary vessels.
Lateral	Lateral edge of palpable/CT-visible gland, anterior to the thoracodorsal artery, and anterior edge of the latissimus dorsi muscle.

Standard	3.2 Tumor Bed and CTV_1
Refer to Standard 2.2 Tumor Bed and CTV_1	

Standard	3.3 Integrated Target Volume CTV_2 for Whole Breast and Low/Mid Axillary
Refer to Standard 2.3 Tumor Bed and CTV_1	
