# Supplementary material for: RecurIndex-Guided postoperative radiotherapy with or without Avoidance of Irradiation of regional Nodes in 1–3 node-positive breast cancer (RIGAIN): a study protocol for a multicentre, open-label, randomised controlled prospective, phase III trial
Source: BMJ Open. 2024 Jul 30;14(7):e078049. doi: 10.1136/bmjopen-2023-078049 (PMC11293409; doi:10.1136/bmjopen-2023-078049)
Supplement: online supplemental file 10 [file bmjopen-14-7-s010.pdf]

Supplementary 10. Prescribed Radiation Dose and Evaluation

I. Purpose: To ensure the smooth conduct of the clinical trial and to guarantee the quality of the clinical trial.

II. Scope: This clinical trial.

III. Procedures:

1. **Prescribed Dose and Fractionation:**

   1.1. RNI + WBI (BCS)/CWI (mastectomy): 5000 cGy in 25 fractions, 200 cGy per day, five days a week. For breast-conserving patients, a boost to the tumor bed is required, sequentially administered after whole-breast irradiation, with a dose of 1000 cGy-1600 cGy in 5-8 fractions, 200 cGy per day. Alternatively, a hypofractionated radiotherapy scheme can be chosen, with a radiation dose of 4000-4256 cGy in 15 to 16 fractions. For breast-conserving patients, a sequential tumor bed boost is performed after completion of whole breast irradiation, as determined by individual center investigators. It can be delivered using conventional fractionation, with a dose of 1000 cGy in 5 fractions at a rate of 200 cGy per day, or by using hypofractionation, with a dose of 798 cGy-1064 cGy in 3 to 4 fractions at a rate of 266 cGy per day. If there are high-risk factors for local recurrence, such as positive surgical margins, close margins, or young age, the radiation dose for the tumor bed boost may be increased to 1400-1600 cGy in 7 to 8 fractions at a rate of 200 cGy per day.

   1.2. RNI omitted, only WBI (BCS), no CWI (mastectomy): 4000 cGy in 15 fractions or 4250 cGy in 16 fractions, 266 cGy per day (hypofractionation), five days a week; or 5000 cGy in 25 fractions, 200 cGy per day (conventional fractionation), five days a week. A boost to the tumor bed is required, sequentially administered after whole-breast irradiation, with a dose of 1000 cGy-1600 cGy in 5-8 fractions, 200 cGy per day.

2. **Dose Distribution and Organs at Risk Limits:**

   2.1. The prescribed dose should cover at least 95% of the PTV in 95% of the target area, with specific dose distribution determined by each center's policy. 2.2. Organs at risk limits should refer to the Quantitative Analyses of Normal Tissue Effects in the Clinic (QUANTEC) standards.

   2.3. For patients with left breast cancer, RNI + WBI (for BCS) / CWI (for mastectomy)

should have a mean heart dose (Dmean) $\leqslant$ 8 Gy, while WBI (for BCS) alone should be limited to a Dmean $\leqslant$ 5 Gy.
