# Supplementary material for: RecurIndex-Guided postoperative radiotherapy with or without Avoidance of Irradiation of regional Nodes in 1–3 node-positive breast cancer (RIGAIN): a study protocol for a multicentre, open-label, randomised controlled prospective, phase III trial
Source: BMJ Open. 2024 Jul 30;14(7):e078049. doi: 10.1136/bmjopen-2023-078049 (PMC11293409; doi:10.1136/bmjopen-2023-078049)
Supplement: online supplemental file 11 [file bmjopen-14-7-s011.pdf]

Supplementary **11** Dose distribution and organ endangerment limits

| Target Volume | Dmax | Dmin |
|---|---|---|
| **Whole Breast PTV_2** | ≤107% | ≥90% |
| **Tumor Bed PTV_1** | ≤107% | ≥90% |
| **Whole Breast and Low-to-Mid Axilla Integrated Target Volume PTV_2** | ≤107% | ≥90% |
| **Chest Wall PTV_CW** | ≤110% | ≥90% |
| **Supraclavicular (±intranodal clavicular) PTV_LN** | ≤110% | ≥90% |
| **Internal Mammary PTV_IMN** | ≤110% | ≥80% |
