# Supplementary material for: RecurIndex-Guided postoperative radiotherapy with or without Avoidance of Irradiation of regional Nodes in 1–3 node-positive breast cancer (RIGAIN): a study protocol for a multicentre, open-label, randomised controlled prospective, phase III trial
Source: BMJ Open. 2024 Jul 30;14(7):e078049. doi: 10.1136/bmjopen-2023-078049 (PMC11293409; doi:10.1136/bmjopen-2023-078049)
Supplement: online supplemental file 12 [file bmjopen-14-7-s012.pdf]

Supplementary 12. Organ dose/volume/impact data for routine split exposures (except where noted): QUANTEC

Organs	Volume	Type of irradiation (partial organ or specially indicated)	Observation index	Dose (Gy) or dose volume parameter	Incidence (%)	Dose volume parameter description
Spinal Cord	Partial spinal cord Thoracic medulla	3DCRT	Spinal cord lesions	Dmax=50	0.02	Includes all spinal cord cross-sections
Pharynx	pharyngeal constrictor muscle	3DCRT	Dysphagia and shortness of breath	Dmean＜50	＜20	
larynx	Total larynx	3DCRT	Edema	Dmean＜44	＜20	No chemotherapy, based on a single study of patients without laryngeal cancer
	Total larynx	3DCRT	Edema	V50＜27%	＜20	
lung	whole lung	3DCRT	Pneumonia	V20≤30%	＜20	Double lung. Slow dose response Without whole lung treatment irradiation
	whole lung	3DCRT	Pneumonia	Dmean=7	5	
	whole lung	3DCRT	Pneumonia	Dmean=13	10	
	whole lung	3DCRT	Pneumonia	Dmean=20	20	
	whole lung	3DCRT	Pneumonia	Dmean=24	30	
	whole lung	3DCRT	Pneumonia	Dmean=27	40	
Esophagus	Whole Esophagus	3DCRT	≥3 Grade acute esophagitis	Dmean＜34	5-20	Contains various dose limiting factors. Seems to be related to dose volume
	Whole Esophagus	3DCRT	≥ grade 2 acute esophagitis	V35＜50%	＜30	
	Whole Esophagus	3DCRT	≥ grade 2 acute esophagitis	V50＜40%	＜30	
heart	Pericardium	3DCRT	pericarditis	Dmean＜26	＜15	Based on individual studies
	Pericardium	3DCRT	pericarditis	V30＜46%	＜15	
	Whole heart	3DCRT	distant cardiac	V25＜46%	＜1	High standards for

			death			assessing security based on predictive models
Liver	Whole Liver - GTV	3DCRT	Typical RILD	Dmean＜30-32	＜5	Exclude patients with existing liver disease or liver cancer
	Whole Liver - GTV	3DCRT	Typical RILD	Dmean＜42	＜50	Patients with liver disease or hepatocellular carcinoma with a Child-Pugh rating of A, but not active hepatitis B, were included as observation indicators
	Whole Liver - GTV	3DCRT	Typical RILD	Dmean＜28	＜5	
	Whole Liver - GTV	3DCRT	Typical RILD	Dmean＜36	＜50	
	Whole stomach	3DCRT	Ulcer	D100＜45	＜7	

QUANTEC: Quantitative Analysis of Illumination Response in Clinically Normal Tissues; 3DCRT: Three-Dimensional Conformal Radiotherapy; GTV: Gross Tumor Volume; RILD: Radioactive Liver Injury; RTOG: Radiation Therapy Oncology Group of Amer
