# Supplementary material for: RecurIndex-Guided postoperative radiotherapy with or without Avoidance of Irradiation of regional Nodes in 1–3 node-positive breast cancer (RIGAIN): a study protocol for a multicentre, open-label, randomised controlled prospective, phase III trial
Source: BMJ Open. 2024 Jul 30;14(7):e078049. doi: 10.1136/bmjopen-2023-078049 (PMC11293409; doi:10.1136/bmjopen-2023-078049)
Supplement: online supplemental file 13 [file bmjopen-14-7-s013.pdf]

Supplementary 13. Research Schedule

Research Phase	Screening	Radiotherapy period				Follow up period										
Visits	1	2	3	4	5	6	7	8	9	10	11	12	13	14	15	
Time	-3M-0	1W	2W	3W	4-7W	6M	12M	18M	24M	30M	36M	42M	48M	54M	60M	In the event of a relapse
Informed consent	×															
Inclusion/exclusion criteria	×															
Frozen/paraffin tissue	×															
Pathology	×[*]															
Tumor markers	×					×	×	×	×	×	×	×	×	×	×	×
Breast + LN ultrasound/MR	×					×	×	×	×	×	×	×	×	×	×	
Mammography	×[*]						×		×		×		×		×	
Chest X-ray/CT	×					×	×	×	×	×	×	×	×	×	×	×
Abdominal ultrasound/CT	×					×	×	×	×	×	×	×	×	×	×	×
Thyroid + LN ultrasound	×					×	×	×	×	×	×	×	×	×	×	
Bone Scan	×[*]						×		×		×		×		×	×
Cranial CT/MRI																×[***]
Demographic features	×															
Medical history	×															
Physical examination	×	×	×	×	×	×	×	×	×	×	×	×	×	×	×	

Upper and lower arm circumference measurement◆	×				×		×		×		×		×		×	
Body weight	×				×		×		×		×		×		×	
ECOG score	×				×		×		×		×		×		×	
Blood count	×**	×	×	×	×	×	×	×	×	×	×	×	×	×	×	×
Liver and kidney function	×**				×	×	×	×	×	×	×	×	×	×	×	×
Infectious disease	×*															
Serum pregnancy test	×**															
EORTC QLQ-C30	×**				×		×		×		×		×		×	
EORTC QLQ-BR23	×**				×		×		×		×		×		×	
Harris	×**						×		×		×		×		×	
Baker	×**						×		×		×		×		×	
CTCAE V4.03	×**	×	×	×	×		×	×	×		×		×		×	
Acute radiation reaction		×	×	×	×											
Late radiation injury							×	×	×		×		×		×	

◆Upper and lower arm circumference measurements: measured at the bilateral metacarpophalangeal joints (mid-metacarpal), at the wrist, and at the distal 10 cm and proximal 15 cm of the elbow epicondyle.

* Within 12 months prior to randomization

** Within 14 days before randomization

*** When cranial symptoms were present
