# Supplementary material for: RecurIndex-Guided postoperative radiotherapy with or without Avoidance of Irradiation of regional Nodes in 1–3 node-positive breast cancer (RIGAIN): a study protocol for a multicentre, open-label, randomised controlled prospective, phase III trial
Source: BMJ Open. 2024 Jul 30;14(7):e078049. doi: 10.1136/bmjopen-2023-078049 (PMC11293409; doi:10.1136/bmjopen-2023-078049)
Supplement: online supplemental file 14 [file bmjopen-14-7-s014.pdf]

Supplementary 14. **Data Management**

1. Source Data Recording

Monitoring personnel will verify source data to confirm that the data entered by authorized personnel at the trial center into the electronic Case Report Form (eCRF), i.e., the source data, is correct, complete, and indeed originated from the source documents.

Source documents (paper or electronic) refer to patient data recorded at the earliest time and include but are not limited to: hospital records, laboratory records, memos, patient-reported outcomes, assessment checklists, data recorded on automated instruments, microfiche, photographic films, X-rays, etc.

Source documents requiring verification of data integrity and validity must not be altered or destroyed and must be retained in accordance with the applicable regulatory retention policies.

For source data verification, investigators and trial institutions must provide monitoring personnel with direct access to relevant source documents and reports for audit purposes and Institutional Ethics Board/Ethics Committee (IEB/EC) review. Trial centers must also allow regulatory authorities to conduct inspections.

2. Use of Computer Systems

When clinical observation results are entered directly into the computerized medical record system of the study center, electronic records may be considered as source documents if the system has been validated according to regulatory requirements for computerized systems adopted in clinical research. Original data should be saved using appropriate computerized data collection systems. If original data requires modification, the system must retain visual inspection audit trails showing the original data and reasons for modification, along with the names and dates of modification.

3. Case Report Forms

This study utilizes electronic Case Report Forms (e-CRFs) for data collection, completed using the Electronic Data Capture (EDC) system specified by the principal investigator. The designated vendor appointed by the principal investigator will provide training to trial centers and a suitable e-CRF completion manual to the research centers.

All e-CRFs are completed by designated, trained personnel at the trial center, and the investigator or designated personnel must review, electronically sign, and date the e-CRFs.

4. Data Quality Assurance

The clinical trial office where the principal investigator is located is responsible for data management for this study, including monitoring/audit of data quality. Clinical research data will be collected via eCRFs using the EDC system. Data entry into the ECD system will be the responsibility of the research center. In case of discrepancies, the clinical trial office where the principal investigator is located will request explanations from the research center, and this process will be electronically resolved within the EDC system.

The sponsor will develop an EDC study quality standards document outlining methods for quality checks on the data.

Data from center laboratories will be sent directly to the principal investigator, who will process and electronically transfer these data according to the standard operating procedures recognized by the

principal investigator for center laboratories.

e-CRFs and correction documents will be retained in the EDC system during the auditing process. The data retained by the principal investigator will be systematically backed up according to standard operating procedures recognized by the principal investigator for the vendor, and records of research data retention will be kept.

5. Independent Data Safety Committee

A Data Safety Committee will be established, consisting of an odd number of members (typically 5), who are independent of the project team and have signed confidentiality agreements. The committee will primarily conduct a review analysis of positive results (subject relapse and metastasis) and understand the actual study results (without statistical analysis) when half of the subjects are enrolled, voting on whether adjustments to the study protocol are necessary
