# Supplementary material for: RecurIndex-Guided postoperative radiotherapy with or without Avoidance of Irradiation of regional Nodes in 1–3 node-positive breast cancer (RIGAIN): a study protocol for a multicentre, open-label, randomised controlled prospective, phase III trial
Source: BMJ Open. 2024 Jul 30;14(7):e078049. doi: 10.1136/bmjopen-2023-078049 (PMC11293409; doi:10.1136/bmjopen-2023-078049)
Supplement: online supplemental file 15 [file bmjopen-14-7-s015.pdf]

Supplementary 15. **Quality Management Plan**

1 Quality Control

1) Qualifications of Study Personnel: All personnel involved in this trial, including investigators, nurses, statisticians, clinical trial observers, etc., must undergo clinical trial training and work under the guidance of senior professionals.
2) Investigators and other personnel involved in the study should fulfill their responsibilities and strictly adhere to the clinical trial protocol, employing standard operating procedures to ensure the implementation of quality control and quality assurance systems.
3) All observed results and findings in the clinical trial should be verified, and quality control must be conducted at every stage of data processing to ensure data integrity, accuracy, authenticity, and reliability.
4) Investigators and other personnel involved in the study should have sufficient time and reliable sources of subjects for conducting the study.
5) All projects involving imaging and laboratory testing should be carried out by units that comply with national standards.
6) Specimens requiring collection in this study should be collected by designated individuals, and follow-up data should be collected and stored by designated personnel.
7) Testing procedures should be conducted according to the specified Standard Operating Procedures (SOPs).
8) When modifications to the study protocol are required, the Ethics Committee should be convened according to SOPs, fully utilizing the Ethics Committee's functions to ensure the protection of subjects' interests.
9) Each participating unit should establish a file folder, save all original materials as required in the protocol, and arrange them in chronological order for verification purposes.
10) Contract research organizations must appoint trained monitors for the study, who should have relevant medical and pharmaceutical backgrounds, and conduct inspections of the research projects according to SOPs (including: pre-trial visits, initiation visits, routine monitoring visits, and end-of-study visits; see "Monitoring Plan").
11) Inspectors should systematically examine clinical trial-related activities and documents to assess whether the trial is conducted in accordance with the protocol, SOPs, and relevant regulatory requirements, and whether trial data are recorded in a timely, truthful, accurate, and complete manner. Inspections should be conducted by personnel not directly involved in the clinical trial.
12) The establishment of inspection work is to ensure that clinical trials are conducted in accordance with the requirements of the protocol, SOPs, and relevant regulations. Contents include: a) How is the clinical research operated? b) Is the implementation in line with the requirements of the study protocol? c) Is the principal investigator effectively and appropriately monitoring the progress of the study? d) How is the quality of the study: whether the study personnel, study centers, and data trial centers adhere to the requirements of SOPs? e) Are the data copied onto the Case Report Form (CRF) consistent with the original data? f) Overall trial quality (identifying the root causes of issues). g) Are study documents present? Are they stored systematically? Are they interpretable (can trial data be reconstructed from study documents)? h) Inspection of monitoring reports attempts to identify quality trends and consult on corrective measures for procedural issues that

have arisen.

13) Regular inspections, preparation of inspection reports, and holding meetings with relevant personnel to discuss issues identified during the inspection.

2 Monitoring Plan

The monitor conducts three types of visits: study initiation visits, routine visits, and close-out visits.

2.1 Study Initiation Visits

Meet with the principal investigator, establish a visit plan, and introduce the monitoring objectives and plans to the investigator. Review includes: training manuals, forms, study protocols, qualifications of participating researchers, and compliance with data management SOPs, etc. If necessary, a start-up meeting can be convened to discuss the protocol and work content with all doctors and other staff participating in the study, clarify each person's responsibilities, explain the SOP requirements for data entry standards, and the preservation of original data.

2.2 Routine Visits

① Before each visit, review the progress of the trial and unresolved issues from previous visits, contact the investigator to confirm the visit date, develop a plan and agenda for this visit, prepare the required documents and items for the visit.

② Meet with the investigator to explain the main tasks of this visit, understand the progress of the trial (subject enrollment status, CRF completion status, informed consent signing status, etc.), and the resolution of problems identified during previous visits.

③ Check and update the investigator's management files, verify the original documents and CRF forms (pay attention to compliance, completeness, consistency with the protocol, discovery, and reporting of SAEs), check trial materials (storage conditions, distribution and recovery records, compliance with protocol requirements).

④ Collect CRF forms.

⑤ Record any issues discovered, discuss and resolve the problems identified during this visit with the investigator, exchange progress and experiences with other research units.

⑥ Store items retrieved, signed informed consent forms, CRF forms, etc., as required.

⑦ Complete the visit report, update various records, track and resolve any issues discovered, and schedule follow-up visit plans.

⑧ SAEs that occur during the clinical trial must be reported to the Ethics Committee within 24 hours.

⑨ Any changes to the protocol, CRF forms, etc., during the trial require approval from the Ethics Committee. Documents to be submitted to the Ethics Committee during the trial include: protocol amendments, informed consent form amendments, SAE reports, recruitment advertisements (if used).

2.3 Close-Out Visits

① Review any outstanding issues from routine visits and confirm their resolution.

② Confirm the visit time, develop a plan and agenda for this visit.

③ Confirm the completeness and updating of the investigator's management files.

④ Confirm that all CRF forms have been collected.
⑤ Confirm the reporting and tracking of SAEs.
⑥ Check the records of the transport, distribution, and retrieval of various materials for the study.
⑦ Discuss and summarize, confirm any outstanding issues and follow-up work, explain the requirements for the preservation of trial-related documents.
⑧ Follow-up work: Complete the trial close-out monitoring visit report, notify the Ethics Committee of the trial's conclusion, continue to track and resolve any outstanding issues, and archive all documents. Documents to be submitted to the Ethics Committee after the trial ends include: trial closure letter, SAEs after trial closure.

3 Data Requirements

Protocol: After careful reading and agreement, the principal investigator must sign and strictly adhere to the protocol implementation.

Clinical Trial Data: All various original clinical trial data should be recorded promptly, truthfully, accurately, and completely, and copies of laboratory test reports should be retained. The principal investigator must retain records and documents of the study implementation process, including eCRFs, informed consent forms, laboratory test results, and radiotherapy plans, for 5 years after the completion or termination of the study, or for a longer period as required by regulatory authorities (whichever is longer). After this time period, the documents may be destroyed in accordance with local regulatory requirements.

4 Study Summary

Once the required total number of cases is reached and verified, the data analysis center performs data analysis. Based on the statistical analysis report, the responsible unit of the clinical trial and participating units write a summary of the clinical trial and sub-center summary table according to the principles of Good Clinical Practice (GCP) clinical trial guidance.

5 Research Funding

The RecurIndex-related testing expenses for this trial are provided by the collaborating party, with specific funding arrangements outlined in a signed contract.

6 Financial Transparency

The principal investigator is required to provide complete and accurate financial information in accordance with Chinese regulations, in order to submit comprehensive and accurate financial statements or disclosure statements to relevant health authorities. The principal investigator is responsible for providing financial information from the beginning to the completion of the study period.
