# Supplementary material for: RecurIndex-Guided postoperative radiotherapy with or without Avoidance of Irradiation of regional Nodes in 1–3 node-positive breast cancer (RIGAIN): a study protocol for a multicentre, open-label, randomised controlled prospective, phase III trial
Source: BMJ Open. 2024 Jul 30;14(7):e078049. doi: 10.1136/bmjopen-2023-078049 (PMC11293409; doi:10.1136/bmjopen-2023-078049)
Supplement: online supplemental file 16 [file bmjopen-14-7-s016.pdf]

Supplementary 16. Statistical Analysis

1. Population Analysis

Full Analysis Set (FAS): Efficacy analysis will be conducted for all cases randomized according to the intention-to-treat (ITT) principle, and analyzed based on their randomized groups, regardless of the actual radiation therapy group received.

Per-Protocol Set (PPS): Subjects from the FAS set will be excluded if they have major protocol violations that could potentially affect the primary efficacy endpoint IDFS analysis. Efficacy evaluation for this study will be conducted for both FAS and PPS sets, with FAS serving as the primary analysis set.

Safety Set (SS): All subjects randomized and receiving at least one session of radiation therapy belong to the safety analysis set. This dataset utilizes actual radiation therapy groups and is used for safety analysis.

2. Demographic and Baseline Characteristics

Descriptive statistical analysis will be conducted for demographic characteristics such as age, gender, height, weight, as well as other baseline features including medical history.

3. Participant Distribution

Descriptive statistical analysis will be performed on participant enrollment status, study completion status, premature study withdrawal, etc. A tabular summary will outline the distribution of participants across different analysis populations.

4. Efficacy Analysis

Efficacy analysis will be based on both FAS and PPS, with FAS serving as the primary analysis set.
Primary Endpoint Analysis: Invasive Disease-Free Survival (IDFS) serves as the primary efficacy endpoint of this study, defined as the time from randomization to the earliest occurrence of invasive cancer local recurrence, distant metastasis, or death, whichever comes first. The occurrence of invasive disease recurrence will be determined by the assessment results obtained by an independent review committee using pathological or imaging examinations. Patients who have not experienced invasive cancer local recurrence, distant metastasis, or death will have their last follow-up date considered as the censoring date. Patients who have not undergone imaging follow-up after baseline will have the randomization date considered as the censoring date. Cox proportional-hazards model will be used for the analysis of the primary endpoint to calculate the Hazard Ratio and its 95% confidence interval, adjusting for stratification factors and other covariates. Additionally, a Cox proportional-hazards model without covariates will be used to support the analysis results of the primary endpoint. Furthermore, the median invasive disease-free survival for both groups (Group A vs. Group B) will be calculated using the Kaplan-Meier (KM) method, including the 95% confidence interval. KM plots will be used to illustrate the trend of IDFS over time.
Secondary Endpoint Analysis: The analysis will compute the Annual Failure Rate (AFR) for each group, along with the corresponding 95% Clopper-Pearson confidence interval. Local recurrence-

free survival (LRFS), distant metastasis-free survival (DMFS), relapse-free survival (RFS), overall survival (OS), disease-free survival (DFS), and breast cancer-specific mortality (BCSM) will be analyzed using the KM method to determine the median values (including the 95% confidence interval). Changes in total scores of EORTC QLQ-C30 and EORTC QLQ-BS23 from baseline will also be summarized.

5. Safety Analysis

Safety analysis will be based on the Safety Set (SS).

The safety analysis will include all enrolled patients who have received at least one session of radiation therapy, grouped according to the actual treatment received by the patients.

Safety will be assessed by summarizing adverse events, changes in laboratory test results, vital sign changes, and exposure to study treatment, reported by treatment group.

All adverse events (AEs) will be classified according to the Medical Dictionary for Regulatory Activities (MedDRA) terminology and graded according to Common Terminology Criteria for Adverse Events (CTCAE) version 4.03, Radiation Therapy Oncology Group/European Organisation for Research and Treatment of Cancer (RTOG/EORTC) Acute Radiation Morbidity Scoring Criteria (1995), and RTOG/EORTC Late Radiation Morbidity Scoring Criteria (1995). All adverse events occurring during treatment (Treatment Emergent Adverse Events, TEAEs, defined as events occurring within 30 days after the end of the last radiation therapy session), TEAEs of Grade 3 or higher, serious adverse events (SAEs), radiation-related SAEs, and TEAEs leading to trial discontinuation will be summarized by system organ class, preferred term, and group in terms of number and percentage. Additionally, the severity and relatedness of TEAEs to radiation therapy will also be summarized by system organ class, preferred term, and group. If a patient experiences the same adverse event multiple times, the maximum reported severity will be used for summarization.

6. Exploratory Studies

Exploratory studies will investigate the relationship between peripheral blood T lymphocyte subsets and the immunomodulatory effects of radiotherapy, as well as the relationship between circulating tumor DNA (ctDNA), circulating tumor cells (CTCs), and distant tumor eradication effects.

7. Interim Analysis

Interim analysis is not planned for this trial.

8. Final Analysis

The final analysis for this study is planned to be conducted when the last enrolled patient completes radiotherapy and reaches a follow-up of 5 years.
