# Supplementary material for: RecurIndex-Guided postoperative radiotherapy with or without Avoidance of Irradiation of regional Nodes in 1–3 node-positive breast cancer (RIGAIN): a study protocol for a multicentre, open-label, randomised controlled prospective, phase III trial
Source: BMJ Open. 2024 Jul 30;14(7):e078049. doi: 10.1136/bmjopen-2023-078049 (PMC11293409; doi:10.1136/bmjopen-2023-078049)
Supplement: online supplemental file 17 [file bmjopen-14-7-s017.pdf]

Supplementary 17. Physical status ECOG scoring criteria

ECOG scoring criteria	Scoring
Mobility is completely normal and does not differ in any way from that before the onset of the disease	0
Can walk freely and perform light physical activities, including general housework or office work, but cannot perform heavier physical activities	1
Able to walk freely and take care of themselves, but have lost the ability to work, and can get up and move around at least half of the time during the day	2
Only partially able to take care of themselves, bedridden or wheelchair bound for more than half of the day	3
Bedridden and unable to care for themselves	4
Death	5
