# Supplementary material for: RecurIndex-Guided postoperative radiotherapy with or without Avoidance of Irradiation of regional Nodes in 1–3 node-positive breast cancer (RIGAIN): a study protocol for a multicentre, open-label, randomised controlled prospective, phase III trial
Source: BMJ Open. 2024 Jul 30;14(7):e078049. doi: 10.1136/bmjopen-2023-078049 (PMC11293409; doi:10.1136/bmjopen-2023-078049)
Supplement: Supplementary file 18 [file bmjopen-2023-078049.reviewer_comments.pdf]

## PEER REVIEW HISTORY

BMJ Open publishes all reviews undertaken for accepted manuscripts. Reviewers are asked to complete a checklist review form (**http://bmjopen.bmj.com/site/about/resources/checklist.pdf**) and are provided with free text boxes to elaborate on their assessment. These free text comments are reproduced below.

## ARTICLE DETAILS

| | |
|---|---|
| **TITLE (PROVISIONAL)** | RecurIndex-Guided postoperative radiotherapy with or without Avoidance of Irradiation of regional Nodes in 1-3 node-positive breast cancer (RIGAIN): a study protocol for a multicenter, open-label, randomized controlled prospective phase III trial |
| **AUTHORS** | Liu, Jing; Tan, Yuting; Bi, Zhuofei; Huang, Suning; Zhang, Na; Zhang, An-du; Zhao, Lina; Wang, Yu; Liang, Zibin; Hou, Yu; Xu, Xiangying; Chen, Jianying; Wang, Fei; Lan, Xiaowen; Lin, Xiao; Zhang, Xiaoxue; Zhou, Wenyi; Ye, Xuting; Ding, Ran; Chen, Jiayi; Huang, Xiaobo |

## VERSION 1 – REVIEW

| | |
|---|---|
| **REVIEWER** | James, Melissa<br>Christchurch Hospital |
| **REVIEW RETURNED** | 04-Sep-2023 |

| | |
|---|---|
| **GENERAL COMMENTS** | 1. Abstract-<br>Would be helpful to mention in the abstract that the Recurrence Index is a multigene assay<br>study will continue for 10 years- this is clarified in the strengths section but the description in the abstract should say that patients will be followed for a minimum of 10 years, I feel that the follow-up period should also be defined, rather than open such that it is analyzed appropriately based on recurrence rates and predicted times of recurrences<br>There are a few instances where syntax could be improved eg:pg5. line 27- not all patients can benefit- should be "will" benefit.<br><br>Other comments:<br>Could some interaction with the PAM 50 predictor of recurrence be considered<br>I feel the lack of radiation quality assurance is a definite concern within the protocol. I am concerned that where no regional irradiation is intended clinicians may treat higher "tangents" and partially include volumes where these are to be excluded by protocol.<br>Page 13, line 35- it is likely some of the benefit is from the prevention of reseeding from recurrences rather than completely due to the abscopal effect.<br>The protocol may benefit from a more specific study of lyphoedema- perhpas using LYMQOL |

| | |
|---|---|
| **REVIEWER** | Umemneku-chikere, Chinyereugo<br>University of York, Centre for Review and Dissemination |
| **REVIEW RETURNED** | 29-Feb-2024 |

| | |
|---|---|
| **GENERAL COMMENTS** | Dear Authors,<br>Thank you for the wealth of knowledge you have planned to contribute to existing knowledge regarding treatment for breast cancer geared towards personalized medicine.<br>The introduction, material and methods, and discussion was clearly explained.<br>However, just a few comments which may need a little bit of clarification.<br>1. What is the rationale for not giving the patients in the control group who underwent mastectomy a CWI (page 7 of 45 line 21 - 25) like their counterpart in the experimental group? Because, this might create some inconsistency, as the RNI is the key tool under evaluation.<br>2. How do you plan to adjust and treat data from crossover patients within the study, should in case such arises? This can be included in the material and method section, if there is a plan for this. |

**VERSION 1 – AUTHOR RESPONSE**

Reviewer#1
Comment 1 Would be helpful to mention in the abstract that the Recurrence Index is a multigene assay.
Response: Thank you for your suggestion, I have added 'Recurrence Index is a multigene assay' in the introduction section of the abstract and highlighted it in red in pg4. line 17.

Comment 2 Study will continue for 10 years- this is clarified in the strengths section but the description in the abstract should say that patients will be followed for a minimum of 10 years, I feel that the follow-up period should also be defined, rather than open such that it is analyzed appropriately based on recurrence rates and predicted times of recurrences
Response: Your suggestions have been extremely helpful to us. It appears our initial statement was not precise enough. Our plan is to complete enrollment within five years, with a follow-up period of 60 months after the last participant completes radiation therapy. We have made the necessary corrections, as follows: on the fifth page, sixth line, and seventh line.

Comment 3 There are a few instances where syntax could be improved eg:pg5. line 27- not all patients can benefit- should be "will" benefit.
Response: Your suggestion is excellent. I have already made the modification to this issue and highlighted it in red font on page 4, line 38.

Comment 4 Could some interaction with the PAM 50 predictor of recurrence be considered
Response: We do believe that considering the interrelation with other multi-gene recurrence risk prediction models for early-stage breast cancer is indeed necessary. However, patients in this study are not required to undergo additional multi-gene recurrence risk prediction models. Additionally, it is regrettable that currently in China, the PAM50 predictor of recurrence is not available for patient testing. However, some patients undergo 70-gene testing to determine the necessity of chemotherapy. If patients have undergone these tests simultaneously, we will conduct exploratory analysis in the future.

Comment 5 I feel the lack of radiation quality assurance is a definite concern within the protocol. I am concerned that where no regional irradiation is intended clinicians may treat higher "tangents" and partially include volumes where these are to be excluded by protocol.

Response: We understand your concerns, and in our actual practice, we rigorously control radiation quality assurance. We have very clear and consistent guidelines for target volume delineation, detailed information of which can be found in Supplementary 10 Guidelines for Target Volume Delineation. The research physicians involved in delineating target volumes for this trial must undergo clinical trial training and work under the guidance of senior professionals, strictly adhering to the clinical trial protocol and employing standard operating procedures to ensure the implementation of quality control and quality assurance systems for the trial.

Comment 6 Page 13, line 35- it is likely some of the benefit is from the prevention of reseeding from recurrences rather than completely due to the abscopal effect.
Response: Your suggestion has made our explanation more comprehensive. We have already made the modifications on Page 13, line 35, and highlighted them in red.

Comment 7 The protocol may benefit from a more specific study of lyphoedema- perhpas using LYMQOL.
Response: Thank you very much for your suggestion. However, since our study has already begun, we currently assess the presence of lymphedema in patients using the method of measuring arm circumference. We will consider using LYMQOL in future studies, but it is not required in this protocol. Once again, we appreciate your input.

Reviewer#2
Comment 1 What is the rationale for not giving the patients in the control group who underwent mastectomy a CWI (page 7 of 45 line 21 - 25) like their counterpart in the experimental group? Because, this might create some inconsistency, as the RNI is the key tool under evaluation.
Response: Your question is very insightful. The omission of CWI in the control group is because, in the majority of international studies on radiation reduction, such as the NSABP51 study, when assessing patients post-mastectomy and with low risk of recurrence, both CWI and RNI are omitted. Our study follows this approach. Additionally, in our hospital, the rate of isolated chest wall recurrence in low-risk N1 patients is very low, with an incomplete statistical rate of 2.3%. Therefore, we exclude CWI and RNI for patients at low risk of recurrence.

Comment 2 How do you plan to adjust and treat data from crossover patients within the study, should in case such arises? This can be included in the material and method section, if there is a plan for this.
Response: Thank you for your reminder. There is no plan for crossover patients in our study. Patients who do not adhere to the prescribed radiation therapy protocol after randomization will be considered as dropping out and will not be included in the analysis.

In conclusion, we thank you for your valuable insights and feedback. We look forward to your continued support in the publication process.

Thank you for your time and consideration.

Sincerely,

Authors,
Manuscript title: RecurIndex-Guided postoperative radiotherapy with or without Avoidance of Irradiation of regional Nodes in 1-3 node-positive breast cancer (RIGAIN): a study protocol for a multicenter, open-label, randomized controlled prospective phase III trial
Manuscript ID: bmjopen-2023-078049

## VERSION 2 – REVIEW

| | |
|---|---|
| **REVIEWER** | James, Melissa<br>Christchurch Hospital |
| **REVIEW RETURNED** | 18-Apr-2024 |

| | |
|---|---|
| **GENERAL COMMENTS** | Thank you for addressing my previous review so comprehensively. I am still concerned that there is no centralized review of radiation quality and independent QA of radiation fields proposed. The quality of radiation has been known to impact trial results and my particular concern here is that those in the no RNI arm may not receive the same chestwall, breast radiation fields as those actually getting radiation as an attempt by the clinician to partially treat the nodes. I feel the lack of centralized review of the radiation plans should be noted as a weakness of the study if it is not possible to add this to the protocol. |

| | |
|---|---|
| **REVIEWER** | Umemneku-chikere, Chinyereugo<br>University of York, Centre for Review and Dissemination |
| **REVIEW RETURNED** | 10-Apr-2024 |

| | |
|---|---|
| **GENERAL COMMENTS** | Thank you for responding to my comments. |

## VERSION 2 – AUTHOR RESPONSE

Reviewer#1
Comment I am still concerned that there is no centralized review of radiation quality and independent QA of radiation fields proposed. The quality of radiation has been known to impact trial results and my particular concern here is that those in the no RNI arm may not receive the same chestwall, breast radiation fields as those actually getting radiation as an attempt by the clinician to partially treat the nodes. I feel the lack of centralized review of the radiation plans should be noted as a weakness of the study if it is not possible to add this to the protocol.
Response: Thank you very much for your valuable feedback. We appreciate your concerns regarding the quality assurance of radiation therapy in our study. We want to assure you that we have stringent requirements for radiation therapy quality. Firstly, we had conducted a questionnaire survey among all participating centers to assess their radiation plans, segmentation techniques, field arrangements, plan aprroving schedule and quality assurance, ensuring they align with the standards required for our clinical research. Only centers meeting these criteria were included in the study. Furthermore, we have established a comprehensive Quality Control Standard Operating Procedure (SOP), as detailed in Supplementary File 15. This SOP outlines our procedures for maintaining quality standards throughout the study. In addition, we have provided detailed requirements for radiation fields in Supplementary File 9 and 10, along with regular random checks on target volume delineation by participating centers. Additionally, patients who undergo a mastectomy are entirely exempt from postoperative regional nodal irradiation (RNI) and chest wall irradiation (CWI); there will be no instances where only the chest wall is irradiated among the enrolled patients. We are committed to upholding the highest standards of quality in our research, and we sincerely appreciate your input to enhance the integrity of our study.

In conclusion, we thank you for your valuable insights and feedback. We look forward to your continued support in the publication process.

Thank you for your time and consideration.

Sincerely,

Authors,
Manuscript title: RecurIndex-Guided postoperative radiotherapy with or without Avoidance of Irradiation of regional Nodes in 1-3 node-positive breast cancer (RIGAIN): a study protocol for a multicenter, open-label, randomized controlled prospective phase III trial
Manuscript ID: bmjopen-2023-078049.R1
